# Multi‐Organ Microphysiological Systems Targeting Specific Organs for Recapitulating Disease Phenotypes via Organ Crosstalk

**DOI:** 10.1002/smsc.202400314

**Published:** 2024-09-19

**Authors:** Joeng Ju Kim, Mihyeon Bae, Dong‐Woo Cho

**Affiliations:** ^1^ Department of Mechanical Engineering Pohang University of Science and Technology (POSTECH) Pohang Kyungbuk 37673 Republic of Korea; ^2^ POSTECH‐Catholic Biomedical Engineering Institute POSTECH Pohang Kyungbuk 37673 Republic of Korea

**Keywords:** biofabrications, biomaterials, disease in vitro models, multi‐organ microphysiological systems, organs on‐a‐chip

## Abstract

Various systemic metabolic diseases arise from prolonged crosstalk across multiple organs, triggering serious impairments in various physiological systems. These diseases are intricate systemic pathologies characterized by complex mechanisms and an unclear etiology, making the treatment challenging. Efforts have been made to develop in vitro models to understand these diseases and devise new treatments. However, there are limitations in reconstructing the causal relationships between diseases and interorgan crosstalk, including the tissue‐specific microenvironment. Alternatively, multi‐organ microphysiological systems (MOMPS) present new possibilities for capturing the complexity of systemic metabolic diseases by replicating human microphysiology and simulating diverse interorgan crosstalk. Controlled interactions and scalable representations of biological complexity in MOMPS offer a more accurate portrayal of organ interactions, enabling the identification of novel relationships between organ crosstalk, metabolism, and immunity. This, in turn, can yield valuable insights into disease mechanisms and drug development research and enhance the efficiency of preclinical studies. In this review, the examples and technical capabilities of MOMPS pathological modeling for various diseases are discussed, leveraging state‐of‐the‐art biofabrication technology of MOMPS. It evaluates the current opportunities and challenges in this field.

## Graphical abstract

Multi‐organ microphysiological systems (MOMPS) replicate human microphysiology and interorgan crosstalk. The precise fabrication of MOMPS requires various elements, including biomaterials, cell sources, accurate organ crosstalk, biofabrication techniques, and humanized design. The MOMPS enhances the understanding of systemic metabolic disease mechanisms, improves drug development, and increases the efficiency of preclinical studies by capturing the complexity of organ interactions and tissue‐specific microenvironments..

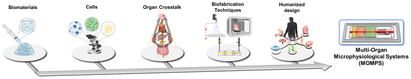

## 1 Introduction

The expansion of the healthcare and pharmaceutical markets and advancements in biomedical engineering have led to active research on the pathological mechanisms of various diseases worldwide.^[^ ^1^ ^]^ Despite extensive research, the pathological mechanisms of complex diseases such as metabolic, neurodegenerative, and autoimmune diseases remain elusive owing to the multifaceted interactions among multiple organs.^[^ ^2^, ^3^ ^]^ This makes it challenging to devise effective prevention and treatment strategies, and the criteria for distinguishing the severity of these conditions remain ambiguous.^[^ ^4^ ^]^ Organ crosstalk and causal relationships among multiple organs can significantly damage various physiological systems and lead to serious health consequences.^[^ ^5^ ^]^ Organ crosstalk generally refers to the biochemical and molecular communication between different organ systems within the human body or between organ mimetics on an in vitro platform.^[^ ^6^ ^]^ This multifaceted interaction involves multiple pathways and feedback loops that can lead to functional changes across organ systems.^[^ ^6^ ^]^ In contrast, a causal relationship refers to a direct cause‐and‐effect mechanism, wherein a change in the function of one organ directly influences a specific outcome in the function of another organ.^[^ ^6^ ^]^ Organ crosstalk and causal relationships frequently involve the exchange of signaling molecules or cytokines, which can lead to observable functional changes in the behavior or physiology of organs.^[^ ^7^ ^]^ Specifically, organ crosstalk and causal relationships involving pathological signaling molecules or inflammatory cytokines can contribute to the onset of disease.^[^ ^8^ ^]^ Therefore, elucidating the precise mechanisms of complex diseases requires accounting for the organ crosstalk and causal relationships among multiple organs as well as developing test models for mechanical verification and drug efficacy testing.^[^ ^9^ ^]^

Conventional approaches such as animal experiments have been widely used to reflect crosstalk among various organs and predict clinical outcomes by observing long‐term physiological phenomena.^[^ ^10^ ^]^ However, ethical issues, high experimental costs, genetic differences between species, and individual animal characteristics prevent the complete translation of experimental animal results to humans, requiring alternative models.^[^ ^11^ ^]^ To address this, combining biomedical engineering with advanced microtechnology, efforts have been made to develop various in vitro models that utilize human cells and replicate the microenvironment of human tissues, thereby implementing the key features of human physiological phenomena.^[^ ^12^ ^]^ In vitro models are broadly classified into 2D and 3D models.^[^ ^13^ ^]^ Although 2D models have been widely produced because of their ease of fabrication and management, they fail to reflect the specific physiological phenomena associated with 3D arrangements and interactions among various cells.^[^ ^14^ ^]^ Hence, 3D in vitro models have been developed to simulate organ development by providing an environment that is biochemically similar to the human body and conducive to cell behavior.^[^ ^15^ ^]^ However, 3D in vitro models targeting a single organ have a limited ability to represent complex diseases arising from interactions among multiple organs.^[^ ^16^ ^]^ Moreover, 3D tissue constructs alone find it challenging to replicate systemic circulations, such as the vascular and lymphatic systems, as well as organ‐specific microenvironments, such as microbial communities.^[^ ^17^ ^]^ To overcome these limitations, a paradigm shift toward in vitro models that can incorporate pathways of organ crosstalk, such as the vasculature, and can be expanded to include various organs is needed.^[^ ^18^ ^]^

**Figure 1:**
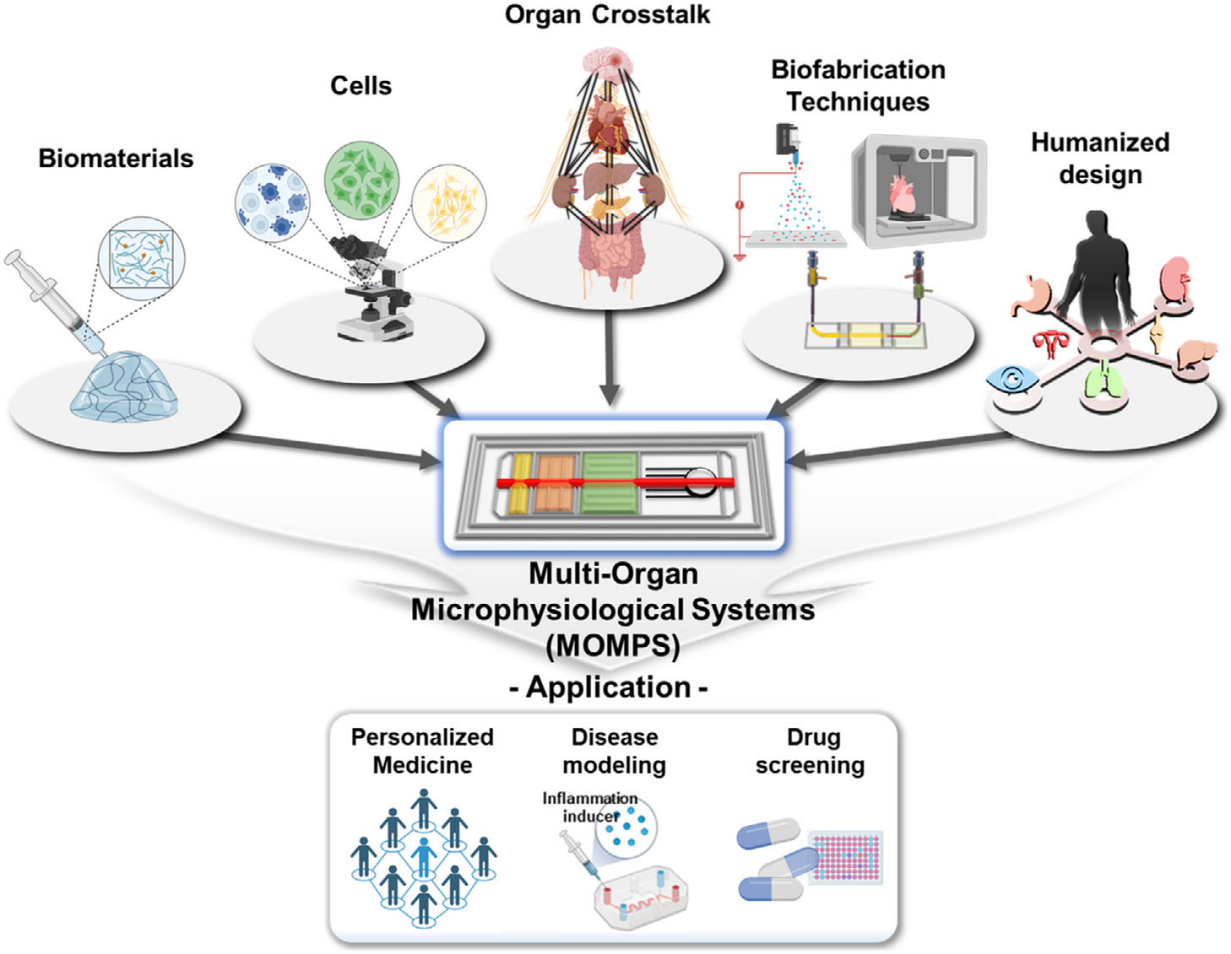
Fabrication aspects and applications of multi‐organ microphysiological systems.

Consequently, advanced models such as multi‐organ microphysiological systems (MOMPS) have been developed to implement interactions among various organs.^[^ ^19^ ^]^ The precise fabrication of MOMPS requires the consideration of various elements, including biomaterials, cell sources, organ arrangement, and interconnection methods for precise organ crosstalk, biofabrication techniques, and humanized design (**Figure** **1**). Reflecting the structural specificity and function of simplified MOMPS by considering organ‐specific cells and the structural characteristics of human organs is essential.^[^ ^20^ ^]^ Additionally, selecting appropriate biomaterials is crucial, as they possess various physical properties depending on their molecular structure and can provide the extracellular matrix (ECM) necessary for the structural support of tissues.^[^ ^21^ ^]^ Conventional manufacturing methods such as self‐assembly and soft lithography have been widely used to fabricate microfluidic devices.^[^ ^22^ ^]^ Recently, more advanced technologies, such as 3D bioprinting and organoid formation, have been increasingly used to create MOMPS.^[^ ^23^ ^]^ Compared to conventional manufacturing techniques, 3D bioprinting offers advantages such as the use of various cell‐friendly biomaterials, freedom to change model designs, and ability to create complex geometries, including vascular structures.^[^ ^24^ ^]^ Therefore, using 3D bioprinting, different cell types can be encapsulated into the platform simultaneously and produced in a short period, thereby enabling the creation of models with higher disease predictability through precise 3D biomimicry.^[^ ^25^ ^]^ Moreover, compared to conventional manufacturing techniques, the organoid formation method accurately represents the functional characteristics of real organs through self‐organization, including multiple cell types within a single organ, and changes in response to stimuli can be observed in real time.^[^ ^26^ ^]^ Additionally, long‐term culture is possible, making it suitable for modeling the progression of chronic diseases.^[^ ^27^ ^]^ However, in the case of 3D bioprinting, the limited resolution may not be sufficient to fully capture the finer details of cellular arrangements or the functional vascular networks that support dynamic processes.^[^ ^7^ ^]^ Additionally, the physical properties of biomaterials used in 3D bioprinting may not accurately replicate the biochemical and mechanical characteristics unique to specific organs.^[^ ^28^ ^]^ These limitations in mechanical properties can influence cell behavior and the diffusion of signaling molecules, thereby hindering the replication of natural organ crosstalk.^[^ ^29^ ^]^ Similarly, with organoid formation methods, the restricted freedom of fabrication may lead to the omission or improper replication of key cellular interactions and microenvironmental factors, resulting in incomplete functional mimicry.^[^ ^30^ ^]^ The diffusion of signaling molecules between organoids may also be inconsistent, potentially hindering effective crosstalk.^[^ ^31^ ^]^ Therefore, to overcome these limitations in MOMPS development, further technological advancements in both 3D bioprinting and organoid formation methods are necessary.

The MOMPSs created using the various biofabrication methods mentioned earlier are advantageous mainly because they integrate and expand multiple organ‐on‐a‐chip technologies, creating a controlled environment to study interactions between different organs.^[^ ^32^ ^]^ By integrating multiple organ‐on‐a‐chip technologies, various hydrogels and cells constituting an organ‐specific microenvironment can be used on a single platform, and a microfluidic platform with precisely fabricated chambers and channels can be produced.^[^ ^33^ ^]^ This allows the simulation of the 3D microenvironment of various organs, and by mimicking the crosstalk between multiple organs, comprehensive system‐level functions can be replicated.^[^ ^20^ ^]^ Miller et al. developed a pumpless MOMPS that integrated compartments simulating up to 14 different human organs. It was designed to model organ crosstalk and simulate drug distribution, metabolism, and systemic effects.^[^ ^34^ ^]^ Their research represented a significant advancement in preclinical drug testing and modeling systemic organ interactions. Consequently, researchers can systematically manipulate variables to observe the effects of abnormalities in one organ system on distant organs through the platform network, providing insights into the causal relationships driving disease progression, and the cellular responses and signaling pathways involved.^[^ ^35^ ^]^ This scalability is crucial for studying systemic metabolic diseases that involve interactions across multiple organs.^[^ ^16^ ^]^ For instance, MOMPS can be used to investigate how inflammatory signals from the adipose tissue affect cardiovascular health, enabling the identification of new relationships between metabolism and immunity, providing valuable insights into the mechanisms of systemic metabolic diseases, and enhancing the efficiency and predictability of preclinical research.^[^ ^36^ ^]^ Slaughter et al. developed MOMPS to model nonalcoholic fatty liver disease (NAFLD) by integrating liver and adipose tissue compartments. It demonstrated significant crosstalk between these organs, with adipokines from adipose tissue exacerbating liver steatosis, replicating key aspects of the NAFLD.^[^ ^37^ ^]^ Additionally, MOMPS provides a more accurate and physiologically relevant model for drug testing, potentially reducing the need for animal models and improving the predictability of human responses.^[^ ^38^ ^]^ McAleer et al. developed a reconfigurable pumpless MOMPS, effectively simulating both the on‐target efficacy and off‐target toxicity of various anticancer drugs, including their metabolites, across multiple organs.^[^ ^38^ ^]^ The ability of their approach to monitor real‐time functional outcomes, such as cardiac electrical and mechanical responses, represents a significant advancement in preclinical drug evaluation, offering a more comprehensive understanding of drug effects across multiple organ systems. Oleaga et al. developed a pumpless MOMPS simulating the liver, heart, skeletal muscle, and neurons to model drug toxicity.^[^ ^39^ ^]^ It demonstrated significant organ crosstalk, particularly showing how liver metabolism of drugs such as doxorubicin could impact cardiac and muscle function. This MOMPS used gravity‐driven fluid flow and microfabricated technologies to maintain organ functionality over extended periods, providing a more accurate alternative to animal models. Furthermore, MOMPS can incorporate patient‐specific cells to create personalized models, allowing the study of individual responses to treatment and the development of customized therapeutic strategies.^[^ ^40^ ^]^ Despite these advantages, precise control of the microenvironment to enable proper interaction among multiple organ systems and the reproducibility required to generate consistent results across models are issues that need to be addressed in advanced MOMPS fabrication.^[^ ^41^ ^]^

This review explains MOMPS created using various biofabrication methods and their application to complex diseases, focusing on organ interactions. The technical capabilities of each case are discussed, and the current opportunities and challenges in this field are evaluated. This approach contributes to a delicate understanding of the interaction effects that occur when multiple organs are involved in the injury and healing processes. The remainder of this article is organized as follows. Section 2 introduces the latest biofabrication technologies and biomaterials used for MOMPS development. Section 3 presents examples of various MOMPS that reflect the pathological mechanisms underlying systemic metabolic diseases. We herein focus on introducing examples of MOMPS related to complex diseases, including neurodegenerative diseases, diabetes, and kidney‐related metabolic disorders in this section. These diseases are prevalent worldwide, yet their pathological mechanisms remain unclear as they usually involve the complex interplay of multiple organs.^[^ ^2^, ^3^ ^]^ Given the current state of MOMPS technology, focusing on brain‐, kidney‐, and diabetes‐related MOMPS is particularly meaningful, as these areas have seen the most significant technological advancements and represent areas of strong clinical needs.^[^ ^7^ ^]^ Additionally, by providing examples of recent MOMPS that target the liver and gut, we aim to offer a more comprehensive analysis that can meaningfully contribute to the understanding of these complex diseases by narrowing the scope to models that focus on specific organs in a balanced manner. Section 4 discusses the current limitations of biofabrication technologies for MOMPS and issues related to MOMPS. Finally, we propose methods to overcome these limitations and strategies for developing MOMPS to simulate a broader range of diseases.

## 2 Latest Fabrication Techniques for MOMPSs

The development of MOMPS relies on the use of advanced biomaterials and biofabrication methods that accurately replicate the complex environments of human tissues.^[^ ^42^ ^]^ MOMPS facilitates the study of organ‐to‐organ crosstalk and promotes the development of new therapies and drugs for systemic metabolic diseases.^[^ ^16^ ^]^ The latest technologies for producing highly functional and physiologically relevant MOMPS employ innovative materials and sophisticated biofabrication methods that enhance the functionality, scalability, and physiological relevance of the models.^[^ ^20^ ^]^ This introduction provides a brief overview of the key materials used in MOMPS fabrication, advanced manufacturing techniques employed, and types of MOMPS products currently available.

### 2.1 Biomaterials for MOMPSs Fabrication

The fabrication of MOMPS and their application as disease models rely heavily on the use of advanced biomaterials capable of mimicking the complex structure and pathological microenvironment of human organs.^[^ ^19^ ^]^ Biomaterials, which are broadly categorized into natural and synthetic materials, are essential for providing the necessary microenvironment for cells and structural support.^[^ ^43^ ^]^ To fabricate MOMPS, biomaterials must possess sufficient mechanical strength and biocompatibility to maintain organ‐specific structures and fluid flow and meet specific requirements such as supporting cell growth and function, printability, and cross‐linking methods.^[^ ^44^ ^]^ Consequently, various hydrogels, biodegradable synthetic polymers, and cell‐containing matrices can be precisely arranged within a single MOMPS to create complex tissue structures for specific purposes.^[^ ^45^ ^]^ This section discusses the characteristics of representative biomaterials widely used in MOMPS fabrication and their applications in disease models, categorized by their intended use. **Table** **1** summarizes the properties of these biomaterials.

**Table 1:** Properties of biomaterials used in fabrication of MOMPS.

Biomaterial	Advantages	Limitations	References
Collagen	Numerous binding sites	Poor physical properties	[50, 52]
	Excellent biocompatibility		
Gelatin	Thermoresponsive behavior	Susceptible to moisture	[57, 58]
Alginate	Controllable mechanical and rheological properties	Poor cell adhesion properties	[61, 62]
SF	Excellent biocompatibility	Long cross‐linking time Low viscosity	[63, 64]
PEG	Excellent biocompatibility	Poor cell adhesion properties	[65, 66]
PVA	High mechanical and tensile strength	Poor cell adhesion properties	[67, 68]
PDMS	Precise control of fluid flow	Low hydrophilicity	[72, 74]
	Excellent biocompatibility and oxygen permeability	Limited use according to biofabrication method	
PCL	Excellent biocompatibility mechanical support	Cell encapsulation not feasible	[78, 79]
PLGA	Excellent biocompatibility mechanical support	Cell encapsulation not feasible	[80, 81]
PF‐127	Thermoreversible gelation behavior	Limited use according to biofabrication method	[82, 83]
dECMs	Various ECM components	Low shape fidelity	[85, 88]
	Powerful tissue specificity		

#### 2.1.1 Hydrogels

Hydrogels are water‐rich cross‐linked polymer networks that mimic the ECM of tissues.^[^ ^46^ ^]^ Hydrogels can be natural or synthetic biomaterials, are highly versatile, and can be processed to possess specific physical, chemical, and biological properties.^[^ ^47^ ^]^ Because of these characteristics, hydrogels are commonly used as bioinks for the 3D bioprinting of living cells and are suitable for creating various forms and biomimetic environments.^[^ ^48^ ^]^

Natural hydrogels, including materials such as collagen, gelatin, fibrin, alginate, silk fibroin (SF), and agarose, are extracted from various organisms and are well suited for simulating tissue microenvironments in MOMPS because of their high biocompatibility and ability to support cell adhesion and proliferation.^[^ ^22^ ^]^ These natural biomaterials are often used as cell‐encapsulation biomaterials in MOMPS because of their high water content and viscoelasticity, which can adequately protect the encapsulated cells.^[^ ^45^ ^]^

Collagen, the most abundant protein in the human body, is a major component of various organs and provides structural integrity owing to its numerous binding sites that promote cell adhesion.^[^ ^49^ ^]^ The properties of collagen, including thermal gelation, biodegradability, and excellent biocompatibility, make it suitable for MOMPS fabrication.^[^ ^50^ ^]^ Collagen Type I is commonly used for MOMPS fabrication and disease modeling, including fibrosis.^[^ ^51^ ^]^ However, collagen exhibits poor physical properties, such as rapid contraction, when used in MOMPS fabrication, leading to deformation of the fabricated structures and low printing resolution.^[^ ^52^ ^]^ Therefore, they are not suitable for manufacturing MOMPS with complex structures or large sizes.^[^ ^53^ ^]^ To overcome these limitations, pure collagen has been chemically modified or mixed with other hydrogels with good physical properties.^[^ ^54^ ^]^ Recently, hybrid collagen‐derived hydrogels with significantly enhanced physical properties were developed by adding photopolymerizable substances, making them useful for the fabrication of complex MOMPS.^[^ ^55^ ^]^

Gelatin, a denatured form of collagen extracted from mammalian tissues, is biodegradable and contains an arginyl–glycyl–aspartic acid (RGD) motif that promotes cell adhesion.^[^ ^56^ ^]^ Because of its thermoresponsive behavior, gelatin is preferred for temperature‐dependent biomanufacturing.^[^ ^57^ ^]^ However, the hydrophilicity of gelatin renders it susceptible to moisture and fluidity at high temperatures, which potentially compromises the structural stability of the fabricated MOMPS.^[^ ^58^ ^]^ To address this issue, gelatin methacryloyl (GelMA), a modified version of gelatin that can be rapidly photo‐cross‐linked with ultraviolet (UV) light, was developed.^[^ ^59^ ^]^ GelMA has dramatically improved printability and structural stability, making it a widely used hydrogel for the fabrication of complex MOMPS.^[^ ^60^ ^]^

Alginate, a polysaccharide extracted from brown algae, is widely used in 3D bioprinting and MOMPS fabrication because of its low cost, controllable mechanical and rheological properties, and immediate cross‐linking reaction with divalent cations, which causes minimal cell damage during cross‐linking.^[^ ^61^ ^]^ However, as alginate lacks ligands for cell attachment, it is often mixed with other hydrogels or chemically modified with binding sites to enhance its biological activity and speed of gelation.^[^ ^62^ ^]^ This approach allows for the fabrication of complex MOMPS structures and improves their structural stability.

The SF, which is derived from silkworms, is frequently used as a biomaterial in tissue engineering owing to its excellent biocompatibility, ease of processing, and high mechanical strength.^[^ ^63^ ^]^ However, pure SF has a long cross‐linking time and low viscosity.^[^ ^43^ ^]^ Therefore, it can be used to fabricate complex MOMPS structures by physically and chemically modifying pure SF or by mixing it with high‐viscosity hydrogels or supplements to create advanced SF‐based hydrogels.^[^ ^64^ ^]^

Conversely, synthetic hydrogels, such as polyethylene glycol (PEG) and polyvinyl alcohol (PVA), are composed of synthetic materials.^[^ ^18^ ^]^ Synthetic hydrogels made from polymers or monomers can be designed for various applications because of their controllable mechanical and chemical properties.^[^ ^55^ ^]^ The PEG is highly biocompatible and suitable for various MOMPS applications because its degradation rate and mechanical properties can be controlled by adjusting its molecular weight and chemical structure.^[^ ^65^ ^]^ Additionally, its nonprotein adhesive properties prevent nonspecific cell adhesion, allowing precise control of cell placement and movement within the MOMPS.^[^ ^66^ ^]^ However, PEG lacks cell adhesion properties, requires the addition of bioactive substances such as RGD peptides, and has a relatively low mechanical strength, making it unsuitable for MOMPS fabrication, which requires structural support.^[^ ^18^ ^]^ The PVA is relatively inexpensive, easy to process, and possesses high mechanical and tensile strengths, making it useful for fabricating various forms of MOMPS requiring structural support.^[^ ^67^ ^]^ Their high water retention capacity and biocompatibility allow cells to grow in physiologically appropriate environments.^[^ ^68^ ^]^ However, PVA lacks cell adhesion properties and requires the addition of bioactive substances or mixing with other hydrogels. Its gelation requires cooling or specific cross‐linkers, which may limit its compatibility with other biomaterials and reduce freedom in MOMPS fabrication.^[^ ^69^ ^]^

#### 2.1.2 Polydimethylsiloxane

Polydimethylsiloxane (PDMS) is a silicone‐based organic polymer created by mixing a curing agent with an elastomer base.^[^ ^70^ ^]^ They can be patterned into various structures using soft lithography or 3D bioprinting, making them suitable for fabricating MOMPS with tubular structures or complex microchannels. This aids in the precise control of fluid flow and can be reversibly adhered to various materials such as plastic or glass, allowing for the construction of multilayer MOMPS.^[^ ^21^ ^]^ Additionally, its excellent biocompatibility and oxygen permeability make PDMS ideal for use as the outer wall of a platform containing hydrogels or culture media, or for cell cultures requiring oxygen enrichment.^[^ ^71^ ^]^ PDMS exhibits low shrinkage after curing and is elastic, flexible, and durable, making it essential for creating dynamic environments and facilitating its integration with micropumps in MOMPS.^[^ ^72^ ^]^ Moreover, its optical transparency allows for the real‐time monitoring of cell behavior and interactions, which is crucial for MOMPS that include optical systems.^[^ ^18^ ^]^ However, when using digital light processing for MOMPS fabrication, the generated oligomers and monomers can inhibit the polymerization of PDMS, limiting its use in biofabrication methods.^[^ ^73^ ^]^ Additionally, the uncross‐linked oligomers in PDMS may be cytotoxic, and the low hydrophilicity of the PDMS surface can hinder cell adhesion.^[^ ^74^ ^]^ To overcome these cell adhesion difficulties, PDMS surfaces are often coated with other hydrogels with good cell‐adhesive properties, such as laminin, collagen, or fibronectin.^[^ ^75^ ^]^ Nevertheless, selecting hydrogels with delayed cross‐linking and significant shrinkage for coating can severely affect the structural integrity of MOMPS; therefore, an appropriate coating hydrogel must be chosen based on the structural design of MOMPS.^[^ ^18^ ^]^

#### 2.1.3 Biodegradable Synthetic Polymers

Biodegradable synthetic polymers exhibit customizable mechanical properties and excellent printability and are designed to degrade into nontoxic byproducts overtime, ensuring consistent quality.^[^ ^76^ ^]^ Despite their lack of bioactivity, they generally possess better physical properties and rigidity than natural biomaterials, making them suitable for use in MOMPS as cell support frameworks that gradually degrade as the tissue matures.^[^ ^77^ ^]^ Additionally, synthetic biomaterials modified to resist degradation are suitable for constructing MOMPS housings.^[^ ^55^ ^]^

Polycaprolactone (PCL) is a United States Food and Drug Administration (USFDA)‐approved biomaterial with low degradation rates and high biocompatibility that is widely used in regenerative medicine and organ‐mimicking in vitro platforms.^[^ ^78^ ^]^ PCL has a lower melting point than other biodegradable synthetic polymers, allowing it to solidify quickly when extruded from a 3D bioprinter nozzle or molded. Therefore, it is suitable for creating scaffolds that directly support cell structures.^[^ ^79^ ^]^ However, owing to its high transition temperature, PCL cannot be used for cell encapsulation because it imposes stress on the cells.^[^ ^18^ ^]^

Poly(lactic*‐co*‐glycolic acid) (PLGA), a copolymer of lactic acid and glycolic acid, is widely used because of its biocompatibility and adjustable degradation rate.^[^ ^80^ ^]^ Additionally, polylactic acid and polyglycolic acid are homopolymers of lactic acid and glycolic acid, respectively.^[^ ^81^ ^]^ These polymers are used because of their predictable degradation profiles and mechanical properties. They provide initial mechanical support to mature organ structures within MOMPS and gradually degrade into natural metabolites overtime.^[^ ^58^ ^]^

Pluronic F‐127 (PF‐127) is a copolymeric biomaterial composed of two hydrophilic poly(ethylene oxide) layers and a hydrophobic poly(propylene oxide) layer.^[^ ^82^ ^]^ PF‐127 exhibited thermoreversible gelation behavior, transitioning from an insoluble to a soluble state in aqueous solutions depending on the temperature.^[^ ^83^ ^]^ It can be easily removed in its liquid state at temperatures below 4 °C. Conversely, PF‐127 forms micelles and transforms into a viscous gel at temperatures above 4 °C.^[^ ^84^ ^]^ These properties make PF‐127 suitable for creating sacrificial parts, such as vascular structures or perfusable proximal tubules and tracheae, in MOMPS.^[^ ^83^ ^]^

#### 2.1.4 Decellularized ECM

Decellularized ECM (dECM) is a biomaterial derived by removing cells from organs while preserving the structure and composition of the ECM.^[^ ^85^ ^]^ Utilizing dECM allows the recreation of organ‐specific microenvironments and essential biochemical signals that are difficult to achieve using single‐component biomaterials.^[^ ^86^ ^]^ This makes dECM applicable for simulating pathological environments.^[^ ^4^ ^]^ Moreover, dECM can be processed into various forms such as powders, hydrogels, sponges, and other formats, depending on the manufacturing purpose, making it a promising biomaterial for tissue engineering and MOMPS fabrication.^[^ ^87^ ^]^ Owing to their significant impact, dECM have been developed for various organs, revealing distinct protein compositions for each organ‐specific dECM.^[^ ^88^ ^]^ The dECM can enhance cell viability, promote cell growth and differentiation, increase tissue‐specific gene expression, and support organ‐specific cellular activities, thereby providing a highly biomimetic environment.^[^ ^85^ ^]^ Additionally, visceral adipose tissue‐derived dECM (vadECM) has been proven to excel in simulating pathological environments for metabolic diseases, including diabetes.^[^ ^8^ ^]^ However, the application of dECM bioink faces challenges such as batch‐to‐batch variability in ECM composition, weak physical properties, low resolution, and potential xenogeneic immune responses.^[^ ^86^ ^]^ To address these issues, recent efforts have focused on automating dECM bioink production and standardizing manufacturing processes to minimize the variability in ECM composition between batches.^[^ ^89^ ^]^ In addition, hybrid dECM bioinks incorporating photopolymerizable materials have been developed to offer superior physical properties, making them suitable for MOMPS and pathological model fabrication.^[^ ^90^ ^]^

### 2.2 Biofabrication Methods for MOMPSs

To construct an MOMPS, it is essential to select an appropriate biofabrication method that considers the specific characteristics of each organ, as well as the interactions between tissues and organs. Advancements in biofabrication technology have provided numerous options for recreating the diverse features of tissues and organs.^[^ ^91^, ^92^ ^]^ These technologies include manufacturing‐based methodologies, such as 3D bioprinting and soft lithography, as well as the generation of organoids that leverage the pluripotency of the cells themselves.^[^ ^14^, ^28^, ^93^, ^94^, ^95^ ^]^ To construct accurate organ models, selecting appropriate cells that correspond to each specific organ and using biomaterials capable of replicating the mechanical stiffness and microstructures of the organs are crucial. However, from a biofabrication perspective, considering how to simulate the organ framework by integrating biofabrication methods, cellular components, and biomaterials is equally important.^[^ ^19^, ^96^ ^]^ In this section, we discuss the characteristics of various biofabrication methods and the precautions necessary for their use, with a focus on accurately simulating the features of human organs.

#### 2.2.1 Organoid Generation

Among various biofabrication methods, organoid generation and culture are promising approaches that recapitulate the functional, structural, and biological complexity of organs through the reproduction and differentiation of pluripotent stem cells (PSCs).^[^ ^26^, ^97^ ^]^ Organoid generation refers to the ability of PSCs to form 3D structures autonomously.^[^ ^98^ ^]^ Therefore, providing a physiological environment conducive to self‐organization is essential.^[^ ^99^ ^]^ Unlike other biofabrication methods, organoid generation relies more on the inherent assembly capabilities of cells than on the engineered design of the production processes. Therefore, developing supporting structures that enhance the ability of cells to effectively self‐assemble is crucial. A representative method for organoid production involves coating well plates (e.g., 96 wells or 384 wells) with a matrix that supports stem cell growth or encapsulates stem cells in a matrix to induce self‐assembly.^[^ ^99^ ^]^ Additionally, to accelerate organoid generation and manipulate the maturation level, it is feasible to apply microfluidic stimuli or microgravity using a bioreactor.^[^ ^100^ ^]^

Among currently developed organoid models, brain organoids are the most representative. Lancaster et al. developed brain organoids and simulated specific regions, as well as the developmental processes of the brain cortex.^[^ ^101^ ^]^ This in vitro model with brain organoids provides insight into the neurodevelopmental process, as well as stepping stones for understanding neurological disorders. Additionally, recent advancements have shown that cerebral organoids can develop retinal structures resembling early‐stage eyes. Remarkably, these optic vesicles respond to light, and the neurons within them exhibit light sensitivity. These findings demonstrate that neural organoids have the potential to emulate not only brain development but also the formation of other organs, highlighting their capacity to mimic complex developmental processes.^[^ ^102^ ^]^ Martins et al. developed a neuromuscular organoid with neuromesodermal progenitor cells.^[^ ^103^ ^]^ The generated organoids included spinal cords and skeletal muscles, providing a foundation for neuromuscular disease research.

In another example, digestive system‐related organoid models, such as those derived from the intestine, liver, and stomach, have garnered significant interest for their potential in advancing the study of digestive diseases.^[^ ^104^, ^105^, ^106^, ^107^, ^108^ ^]^ These organs are particularly susceptible to external factors, including diet, alcohol consumption, and bacterial infections. Thus, the response of these digestive organs to such exogenous factors must be studied based on their distinct physiological structures. For example, the liver plays a vital role in the body regarding detoxication and metabolism with enzymes. This organ is strongly affected by drug toxicity; thus, liver organoids have been recommended for studying liver toxicity.^[^ ^109^, ^110^ ^]^ Further, intestinal organoids can replicate the intricate architecture of the native intestine, including crypts and villi.^[^ ^26^, ^111^ ^]^ The presence of these structures in intestinal organoids offers a key advantage: the ability to model patient‐specific genetic characteristics, such as fibrosis and epidermal disruption during inflammation. This makes them an invaluable tool for personalized disease modeling and therapeutic testing.

Organoids are being increasingly utilized as cancer models.^[^ ^112^, ^113^ ^]^ A major challenge in treating cancer is the patient‐specific chemoresistance, which includes factors such as cancer cell viability and growth rate. Therefore, the use of patient‐derived cells is crucial to identifying the most effective treatment strategies. In this context, cancer organoids are particularly advantageous for simulating cancer physiology, as they maintain both genetic and phenotypic fidelities. For instance, gastric cancer organoids can be constructed using patient‐derived gastric cancer cells, effectively recapitulating tumor heterogeneity through the inclusion of diverse cell populations.^[^ ^114^ ^]^ Additionally, these organoids can replicate the complex inner structure of gastric cancer through cellular assembly. Consequently, cancer organoids provide a valuable platform for summarizing the pathophysiological features of cancer, particularly solid tumors, while capturing the genetic variability that is critical for personalized medicine.

Organoid‐based in vitro organ models have the advantage of simulating a broad range of disease phenomena. Several factors must be considered to build a more reliable organoid model. The quality of the cell source is a key factor in organoid generation. Although embryonic stem cells are highly pluripotent, their use is limited because of ethical concerns. Consequently, other PSCs or induced PSCs (iPSCs) are typically used as alternatives.^[^ ^115^, ^116^ ^]^ Furthermore, the viability and genetic stability of these cells significantly affect the similarity of organoids to native organs, as well as the yield and integrity of organoids. Therefore, determining appropriate growth factor combinations as stabilized protocols is necessary to ensure high maturation and stable growth of stem cells for organoid formation.

#### 2.2.2 Soft‐Lithography‐Based Biofabrication

Soft‐lithography‐based biofabrication is commonly used to produce microfluidically adapted structures.^[^ ^117^ ^]^ In this method, a mold for the organ model is created by hardening PDMS onto a pre‐built pattern on a substrate.^[^ ^118^ ^]^ This technique offers several advantages, including the ability to construct microscale channels for microfluidic applications, flexibility in creating various channel patterns, and the capability for mass production, such as high‐throughput screening.^[^ ^119^ ^]^ To perform soft lithography, photolithography is first employed to create a patterned substrate. Photolithography is a fabrication method that involves depositing photoresist materials onto a substrate such as a silicon wafer and then etching the desired pattern.^[^ ^120^ ^]^ After the patterned substrate is created, an elastomer such as PDMS is poured onto the substrate to produce microstructures that recapitulate the organ structure. This process enables the precise fabrication of the microscale features necessary for organ model construction. In addition, the surface of the patterned elastomer chamber can be modified for hydrophilicity using plasma treatment to facilitate cell attachment within the channels.^[^ ^121^ ^]^ The chambers can also be sterilized using UV treatment.^[^ ^14^ ^]^ When constructing a soft‐lithography‐based MOMPS, considering the oxygen gradient is important because PDMS is gas permeable. Furthermore, PDMS tends to adhere to proteins and small organic molecules owing to its hydrophobic nature. To prevent this, the surfaces of soft‐lithography‐based chips constructed with PDMS should be coated with PEG.^[^ ^122^, ^123^, ^124^ ^]^ Moreover, an appropriate mixture of the elastomer and cross‐linker should be carefully considered to achieve the desired physical properties for the designed organ because the stiffness of the hardened PDMS depends on the concentration of the cross‐linker in the elastomer.^[^ ^125^ ^]^

The MOMPS technology originated from soft‐lithography‐based microfluidic chips. The cells were injected into the microfluidic channels and controlled by the microfluidic flow. This soft‐lithography‐based MOMPS has revolutionized the paradigm of in vitro organ models, offering the advantage of conducting analyses with small sample sizes because of the model's ability to replicate organ behavior on the microscale. Since the development of the lung‐on‐a‐chip model by Huh et al. in 2010, various other organ‐on‐a‐chip models have been developed, including heart‐on‐a‐chip and eye‐on‐a‐chip models.^[^ ^126^, ^127^, ^128^ ^]^ Moreover, MOMPS, such as those integrating heart‐on‐a‐chip with other organs, have been proposed to assess cardiotoxicity. Recently, researchers simulated infectious diseases using soft‐lithography‐based lung‐on‐a‐chip models.^[^ ^129^, ^130^ ^]^ The advantages of microfluidics‐based models, such as the requirement for only small amounts of samples, provide a platform for rapidly studying disease behavior and testing drugs.

As such, soft‐lithography‐based MOMPS has led to a significant paradigm shift in disease research; however, it can be further improved with the following considerations. First, a 3D environment needs to be constructed within the soft‐lithography‐based MOMPS. Although the microfluidic environment can simulate microenvironments, the behavior of organs on a submillimeter scale, such as blood flow in arteries or liver cell behavior in sinusoidal structures, is limited in the current soft‐lithography‐based MOMPS. Thus, to accurately represent scale‐ and structure‐specific cell behaviors, it is necessary to build channels of varying structures and sizes within the soft‐lithography‐based MOMPS. This approach allows for a more precise simulation of the complex environments found in organs.

#### 2.2.3 3D Bioprinting

The 3D bioprinting is a type of additive manufacturing known as 3D printing.^[^ ^28^ ^]^ This method involves building 3D tissue analogs using cells and biomaterials as “ink”.^[^ ^50^ ^]^ Currently developed in vitro tissue models incorporate 3D bioprinting, which offers several advantages. One of its primary benefits is the flexibility to produce a wide variety of tissues and organs by combining different types of cells and biomaterials, allowing engineers to design and construct complex biological structures according to their specific intentions.^[^ ^131^ ^]^ The versatility of 3D bioprinting can be realized using different printing techniques, such as extrusion‐based, inkjet, and laser‐assisted bioprinting.^[^ ^18^, ^52^ ^]^ Each technique offers distinct benefits and drawbacks. For example, while extrusion‐based bioprinting allows for a broad selection of materials, it is hindered by slower processing times. Inkjet bioprinting is known for its rapid speed and cost‐effectiveness, but it falls short in terms of material variety and precision.^[^ ^132^, ^133^ ^]^ Laser‐assisted bioprinting, although capable of achieving high cell viability, requires complex equipment. Consequently, the choice of printing technique should align with the specific goals and priorities of the bioprinting project, as it significantly influences the adaptability of the resulting bioprinted structure.

Several manufacturing factors should be considered when adopting 3D bioprinting to build an organ model. First, the printability of the biomaterials is crucial. For example, extrusion bioprinting, the most commonly used 3D‐bioprinting method, requires pneumatic or mechanical pressure to print a material.^[^ ^52^ ^]^ If the viscosity of the biomaterial is too high, it can clog the nozzle, resulting in an inhomogeneously printed structure and decreased cellular viability. Therefore, printable biomaterials should possess shear‐thinning properties, in which the viscosity of the material decreases linearly as the pressure increases.^[^ ^134^ ^]^ For laser‐assisted bioprinting, it is essential to choose an appropriate photocross‐linker that reacts properly to the light source and is not toxic to the cells.^[^ ^135^ ^]^ In summary, the physical properties of the biomaterials should match the desired bioprinting method to ensure successful printing and cell viability. Additionally, cellular apoptosis should be prevented, as it can be influenced by the manufacturing time and surrounding environment (e.g., temperature). Unlike other biofabrication methods, such as organoid generation and soft lithography‐based biofabrication, 3D bioprinting involves longer contact times between the cells and air. Therefore, it is essential to provide an appropriate surrounding environment that maintains cellular viability by ensuring proper humidity and temperature as well as preventing contamination through sterilization.^[^ ^24^, ^85^ ^]^

The 3D‐bioprinted organ models offer the advantage of simplifying the fabrication process and recapitulating complex organ structures. For instance, Yi et al. developed a 3D‐bioprinted glioblastoma (GBM) model that mimics the physiological structure of GBM, including pseudopalisade formation.^[^ ^136^ ^]^ This model accurately replicates the hypoxic environment typical of solid cancers such as GBM. Using patient‐specific cell sources, 3D‐bioprinted cancer environments can mirror the clinical responses of patients to chemotherapy and radiotherapy, providing a valuable tool for personalized medicine with a simplified fabrication procedure. In addition, by leveraging the advantages of 3D bioprinting to create various shapes, diverse vascular structures have been built to study vascularized diseases.^[^ ^22^, ^137^ ^]^ These vascular models can simulate atherosclerosis and demonstrate different pathophysiologies based on vessel shape.^[^ ^137^ ^]^ Moreover, vascular models can replicate complex structures, such as vessels and biliary tracts, within organs.^[^ ^138^ ^]^ To further enhance the advantages of 3D bioprinting in creating more complex models, developing innovative biomaterials that offer printability, structural integrity, and biocompatibility is crucial.^[^ ^139^ ^]^ For example, various photocross‐linkers such as ruthenium‐sodium persulfate and advanced materials such as vitrified dECM have been introduced to enhance structural integrity and mimic native organs more closely.^[^ ^85^, ^89^, ^135^, ^140^ ^]^

Thus far, we have introduced fabrication methods and representative examples of organoids, soft‐lithography‐based organ models, and 3D‐bioprinted models. Each fabrication method has its own advantages and limitations, and certain aspects cannot be fully addressed using a single method. By combining these methods to create MOMPS, producing more reliable human‐like structures with a high degree of processing freedom is possible. This collaborative approach leverages the strengths of each method to enhance the overall fidelity and functionality of the resulting organ models.^[^ ^112^, ^141^, ^142^ ^]^ **Table** **2** summarizes the advantages and disadvantages of each fabrication method.

**Table 2:** Summary of key characteristics and expected improvement point of each fabrication method.

Fabrication method	Key characteristics	Expected improvement point	References
Organoids generation	–Self‐organization‐based tissue analog growth –High physiological similarity with native organs in the context of developmental process	–Stabilized cell sources to compensate pluripotency rather than embryonic cells –Ensured culture protocol for high maturation and stable growth for organoids	[98, 99]
Soft‐lithography‐based biofabrication	–Simulation of microfluidic flow in physiological system –Enabling high‐throughput assessment due to its microfluidic scale control	–Advanced 3D environment for simulating structural physiology	[119, 120]
3D bioprinting	–High degree of freedom for generating varying tissues and organs with various combination of cells and biomaterials –Varying 3D structure for matching each organ	–Improved printability of biomaterials for structural integrity –Appropriate surrounding fabrication environment for preventing contamination	[131, 139]

## 3 Application of MOMPSs to Simulate Various Systemic Metabolic Diseases

With current technology, it remains impossible to create disease models that perfectly mimic the functions and pathological mechanisms of human organs. This is a major obstacle in the development of MOMPS.^[^ ^19^ ^]^ Nonetheless, the construction of disease models using various functional biomaterials and advanced biofabrication techniques is actively progressing, enabling the modeling of complex interactions between various organs.^[^ ^33^ ^]^ Through these systems, researchers can simulate and analyze the multifaceted characteristics of metabolic disorders, thereby providing insights into disease mechanisms, drug effects, and potential therapeutic interventions.^[^ ^16^ ^]^ This section explores the application of MOMPS in simulating systemic metabolic diseases by connecting organs involved in simultaneous interactions during disease development, focusing on brain‐, kidney‐, and diabetes‐targeted systems. **Table** **3** summarizes the types and characteristics of MOMPS in each organ disease.

**Table 3:** Summary of various MOMPS for simulating systemic diseases.

Target disease	Target organ	Biomaterials	Fabrication method	References
Air‐pollutant‐induced neurodegenerative disease	Neurovascular unit	Matrigel and laminin for cell culture, PDMS for chip modeling	Soft‐lithography‐based biofabrication and self‐assembly	[155]
Parkinson's disease	Gut–liver–brain	Matrigel for cell culture, polycarbonate for chip modeling	Organoid generation	[160]
Oxidative kidney injury	Liver–kidney	PDMS	Self‐assembly	[183]
Secondary hyperoxaluria	Intestine–kidney	Kidney‐derived dECM, poly(ethylene‐co‐vinyl acetate), PF‐127	Extrusion‐based 3D bioprinting	[177]
Diabetes	Pancreas–liver	PDMS	Self‐assembly	[197]
Diabetes	Pancreas–visceral adipose tissue–liver–retina	Pancreas, adipose tissue, and liver‐derived dECMs, PDMS, PCL	Extrusion‐based 3D bioprinting	[18]

### 3.1 Brain Targeted MOMPSs

The brain, the most important organ in the central nervous system, controls the behavior of tissues and organs and receives feedback signals from other organs. Notably, researchers have revealed that the physiological behavior of the brain is influenced by the lungs and gut, which are directly exposed to external substances, such as dust and food. In this section, we introduce the brain‐connected MOMPS and discuss their contributions and areas for improvement.

The brain is primarily composed of the neurovascular unit, which includes diverse populations of neural cells such as neurons, astrocytes, and the specialized vascular structure known as the blood–brain barrier (BBB).^[^ ^143^, ^144^ ^]^ The BBB plays a vital role in safeguarding the brain by restricting the entry of foreign substances. Owing to its selective permeability, the BBB has become a critical focus in the development of drug delivery systems targeting the brain, as this barrier presents a significant obstacle to efficient drug transport. Additionally, compromised BBB permeability is linked to the onset of various neurological disorders, including neurodegenerative diseases. To investigate these mechanisms, in vitro models of the BBB have been established.^[^ ^145^, ^146^ ^]^ These models have been frequently constructed using microfluidic channels within PDMS molds or 3D‐printed vessels. Such BBB Mmicrophysiological systems (MPSs) have been employed to explore the physiological properties of the BBB, including barrier functions mediated by tight junctions between brain endothelial cells and pericytes, as well as disease‐related pathophysiologies such as brain tumors and neuroinflammation due to vascular dysfunction.

In addition to developing models of neural vascular systems, the basic physiological unit of a neural tissue, which is composed of various neural cell populations, should be considered. These populations typically include neurons, astrocytes, and microglia. Neurons are responsible for storing and transmitting neural signals, thereby regulating bodily functions. Astrocytes and microglia, which function similar to immune cells, protect neural tissues. This function is distinct from that in other tissues. Several methods exist for simulating neural cell populations in vitro, including neurospheres, neural organoids, 3D‐bioprinted neural tissues, and microfluidic triple culture systems.^[^ ^147^, ^148^, ^149^, ^150^, ^151^, ^152^ ^]^ These MPSs offer valuable insights into the physiological structure of the brain by enabling the modeling of neurodevelopmental processes, neurodegenerative pathologies, and drug efficacy in a high‐throughpu*t* testing platform. Consequently, the development of MPSs for neural cell populations and the BBB lays the foundation for a deeper understanding of the complex interactions within the brain.

**Figure 2:**
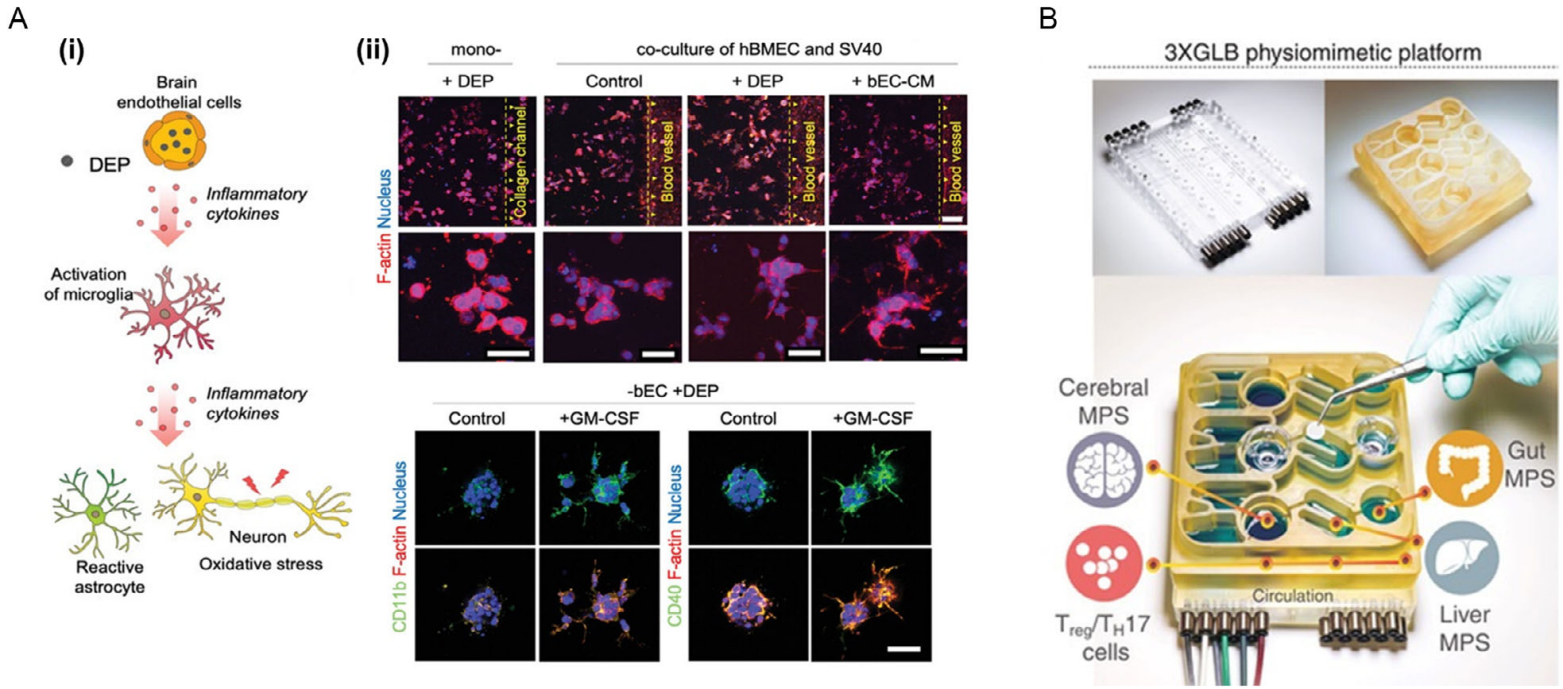
Brain‐based MOMPS. A) Neurovascular unit MOMPS for recapitulating air‐pollutant‐induced neurodegenerative disease. i) Schematic images of pollution‐induced neurodegeneration. ii) Immunofluorescence results for microglia activation with pollutant‐induced cytokine from dysfunctional blood–brain barrier (BBB). Reproduced with permission.^[^ ^155^ ^]^ Copyright 2023, John Wiley and Sons. B) Gut–liver–brain MOMPS designed schematic. Reproduced with permission.^[^ ^160^ ^]^ Copyright 2021, The American Association for the Advancement of Science.

Building on the development of single‐neural MPSs, these models have been integrated with other tissue systems to investigate crosstalk between the brain and other organs.^[^ ^147^, ^153^ ^]^ An example of this is the MOMPS connecting the brain and lung tissues, which has been utilized to study the impact of air pollution on the progression of neurodegenerative diseases. Air pollution has become a serious social and health problem as it worsens with industrial development. Fine particulate matter (PM2.5) can penetrate the BBB through respiratory organs, compromising the primary defense against foreign bodies infiltrating the brain.^[^ ^154^ ^]^ Once these pollutants penetrate the BBB, they can affect immune reactions in the brain and induce neurological disorders. Seo et al. demonstrated that diesel exhaust microparticles that penetrate the BBB can induce neurodegenerative diseases (**Figure** **2**A). They developed a neurovascular unit‐on‐a‐chip using PDMS, which included neural cells and BBB vascular structures, to simulate the basic unit of the brain.^[^ ^155^ ^]^ By injecting air pollutant particles into the BBB's vascular structure, they revealed that these particles could destroy the selective permeability of the BBB, thereby compromising its protective function. Subsequently, they treated microglia, which are the main immune cells in the brain, with cytokines derived from a dysfunctional BBB. Microglia exhibit an M1 phenotype, which is indicative of an immune reaction and neurotoxicity, demonstrating microglial activation due to air pollutants. Furthermore, they explored therapeutic approaches to suppress microglial activation and found that such suppression could be key to slowing neurodegeneration. This vascular–neural tissue combination model suggests the importance of considering neural cell–vascular cell crosstalk in response to air pollution to prevent neurodegenerative diseases. Additionally, these in vitro models can be adapted for drug screening of neurodegenerative diseases.

Another organ system that affects the brain is the digestive system, which includes the gut and intestines. The concept of the gut–brain axis (GBA) has gained attention, highlighting the crosstalk between the central and enteric nervous systems.^[^ ^156^, ^157^ ^]^ This concept implies that the brain not only controls gut behavior but also engages in bidirectional communication with the intestinal microbiome. GBA involves several pathways including immune effects mediated by cytokine transduction, vagus neural signals from the intestine to the brain, and neurosecretion routes for transmitting neurotransmitters and gastrointestinal hormones.^[^ ^158^ ^]^ Understanding GBA has contributed to uncovering the interconnections between neurological disorders, specific microorganisms, and hormonal changes. However, existing animal models are limited in their ability to elucidate clear gut–brain interactions, owing to other interconnected systems beyond the GBA. Therefore, it is necessary to isolate GBA from other organ crosstalks and develop a quantitative analysis platform to clarify the signals within GBA.^[^ ^159^ ^]^ Based on these unmet needs, an in vitro GBA model is needed to recapitulate the crosstalk between the intestinal microbiome and central nervous system. Trapecar et al. developed a gut–brain–liver‐connected MOMPS model to investigate the occurrence of Parkinson's disease resulting from immune reactions to metabolic products (Figure 2B). Parkinson's disease is significantly influenced by the microbiome and the systemic immune responses within the body. Therefore, researchers have designed gut/microbiome–liver–brain models.^[^ ^160^ ^]^ They constructed a gut organoid compartment, a liver compartment to capture metabolites from the gut, and a circulating flow of immune cells to simulate immune transduction to the brain. They demonstrated that the gut/liver and brain interactions enhanced the maturation of neural cells. Furthermore, they found that short‐chain fatty acids in metabolites increased the pathways associated with the pathophysiology of Parkinson's disease under disease conditions, but not in healthy conditions. These findings illustrate that GBA models, which also reflect metabolic processes in the body, can reveal the mechanisms underlying the occurrence of neurodegenerative diseases.

In addition to the previously mentioned examples, the MOMPS connecting neural tissues with muscles have been developed. One of the most severe neuromuscular diseases is amyotrophic lateral sclerosis (ALS), which results from the loss of motor neurons in the brain and spinal cord that control muscle function. As the disease progresses, ALS patients gradually lose their motor abilities, which ultimately leads to their inability to breathe independently.^[^ ^161^, ^162^ ^]^ Despite extensive research, the causes and mechanisms underlying ALS are not fully understood. This has warranted the development of platforms that can explore the physiological interactions between the central nervous system and muscular tissues.^[^ ^163^, ^164^ ^]^ Osaki et al. introduced a neuromuscular MOMPS that incorporates muscle cells and motor neuron spheroids derived from human iPSCs. They constructed 3D skeletal muscle fibers and motor neuron spheroids within a compartmentalized PDMS chamber designed to simulate the neuromuscular junction.^[^ ^165^ ^]^ This engineered system not only demonstrated the crosstalk between motor neurons and muscles via neurotransmitter signaling but also provided insights into ALS pathogenesis, particularly the effects of glutamate‐induced excitotoxicity. Moreover, the study highlighted the model's potential for therapeutic research. By applying rapamycin and bosutinib—both inhibitors of muscle contraction—they demonstrated that the combination of these drugs could ameliorate abnormal muscle contractions associated with ALS. Thus, the neuromuscular MOMPS is a valuable tool for investigating ALS mechanisms and exploring potential drug therapies.

As such, brain‐connected MOMPSs have proven to be useful tools for uncovering the occurrence of neurological diseases resulting from physiological crosstalk with other tissues. Specifically, the onset of neurological disorders owing to interactions with organs that react to foreign matter and neurotransmitter signaling, such as lung–brain, gut–brain, and neuromuscular interactions, can be reconstructed using these brain‐connected MOMPSs.^[^ ^166^, ^167^, ^168^ ^]^ These models enable an understanding of neural cell behavioral changes and the mechanisms of neurological disorders in response to environmental factors, such as air pollution, diet, and alcohol consumption. The following should be considered to create more reliable brain‐connected MOMPSs. First, it is important to select appropriate biomaterials that accurately mimic the physiological environment of the brain and other tissues.^[^ ^169^, ^170^, ^171^ ^]^ Neural cells exhibit varying differentiation behaviors in response to the biophysical properties of their surrounding environment. Additionally, the maturation level of neural cells should be tailored to the target disease because neural cell phenotypes differ between developmental and degenerative processes. For example, to effectively recapitulate neurodegeneration, it is crucial to use sufficiently mature neurons that accurately represent the pathophysiology of degeneration.^[^ ^172^, ^173^ ^]^ A brain MOMPS that accurately reflects these neurological characteristics could be used in the future to identify interactions with a wider range of other organs. These advancements would enhance our understanding of complex physiological interactions and contribute to the development of effective treatments for neurological disorders.

### 3.2 Kidney‐Targeted MOMPSs

Renal diseases involve various conditions that impair the ability of the kidneys to filter blood, remove waste products, and maintain fluid and electrolyte balance.^[^ ^174^ ^]^ Renal diseases can have an extensive impact on other organs and systems of the body.^[^ ^175^ ^]^ Examples of complex diseases involving the kidney and other organs include cardiorenal syndrome, hepatorenal syndrome, intestinal oxalate malabsorption‐related kidney stone disease, and diabetic nephropathy.^[^ ^176^, ^177^, ^178^ ^]^ Cardiorenal syndrome refers to conditions in which decreased cardiac blood flow and pressure lead to kidney damage. Conversely, kidney fluid overload and increased blood pressure cause cardiac damage.^[^ ^176^ ^]^ Hepatorenal syndrome involves renal failure due to changes in blood flow and hormonal imbalances resulting from impaired liver function.^[^ ^179^ ^]^ Therefore, studying not only the microenvironment and pathological features of the kidneys but also the pathological conditions of related organs is crucial to fully understand renal disease mechanisms and develop treatments.^[^ ^22^ ^]^ This requires the development of accurate in vitro kidney disease models that can replicate the pathological features and microenvironment of multi‐organ kidney diseases and enable the study of complex kidney disease mechanisms and therapeutic testing.

Most in vitro kidney models and kidney disease studies have been conducted using 2D cell culture models. However, 2D cultures have limitations in reproducing the 3D structure and functional interactions of the kidneys.^[^ ^180^ ^]^ To create a successful in vitro kidney model, it is necessary to replicate the complex structure and physiological functions of the nephron and kidney filtration units, including the glomerular filtration barrier and tubular reabsorption processes. This requires the incorporation of both kidney epithelial and endothelial cell layers within a single platform and precise control of the microenvironment, including fluid flow and shear stress.^[^ ^181^ ^]^ Consequently, 3D in vitro kidney models have been developed. Functional bioinks capable of reflecting the kidney microenvironment have been developed, and coaxial 3D‐bioprinting technology has been used to create hollow microfluidic tubes within the platform consisting of kidney tubular epithelial and endothelial cells to simulate the reabsorption process.^[^ ^22^ ^]^ Another approach involves using kidney organoids to create a 3D kidney model. Li et al. developed a kidney model for the polycystic kidney disease by combining kidney organoid technology with a microfluidic system.^[^ ^182^ ^]^ This system integrated a microfluidic device to simulate the in vivo environment of the kidney. Thus, it successfully replicated the mechanism by which glucose exacerbates cyst formation. However, most in vitro kidney models, including these examples, are single‐organ models that do not reflect crosstalk with other organs. To accurately replicate kidney diseases, it is essential to use biomaterials that can replicate disease‐specific pathological microenvironments and simulate crosstalk between multiple organs within a single platform.^[^ ^180^ ^]^ The superiority and fabrication technologies of advanced kidney disease‐mimicking MOMPS that satisfy these requirements have been explained through various examples.

**Figure 3:**
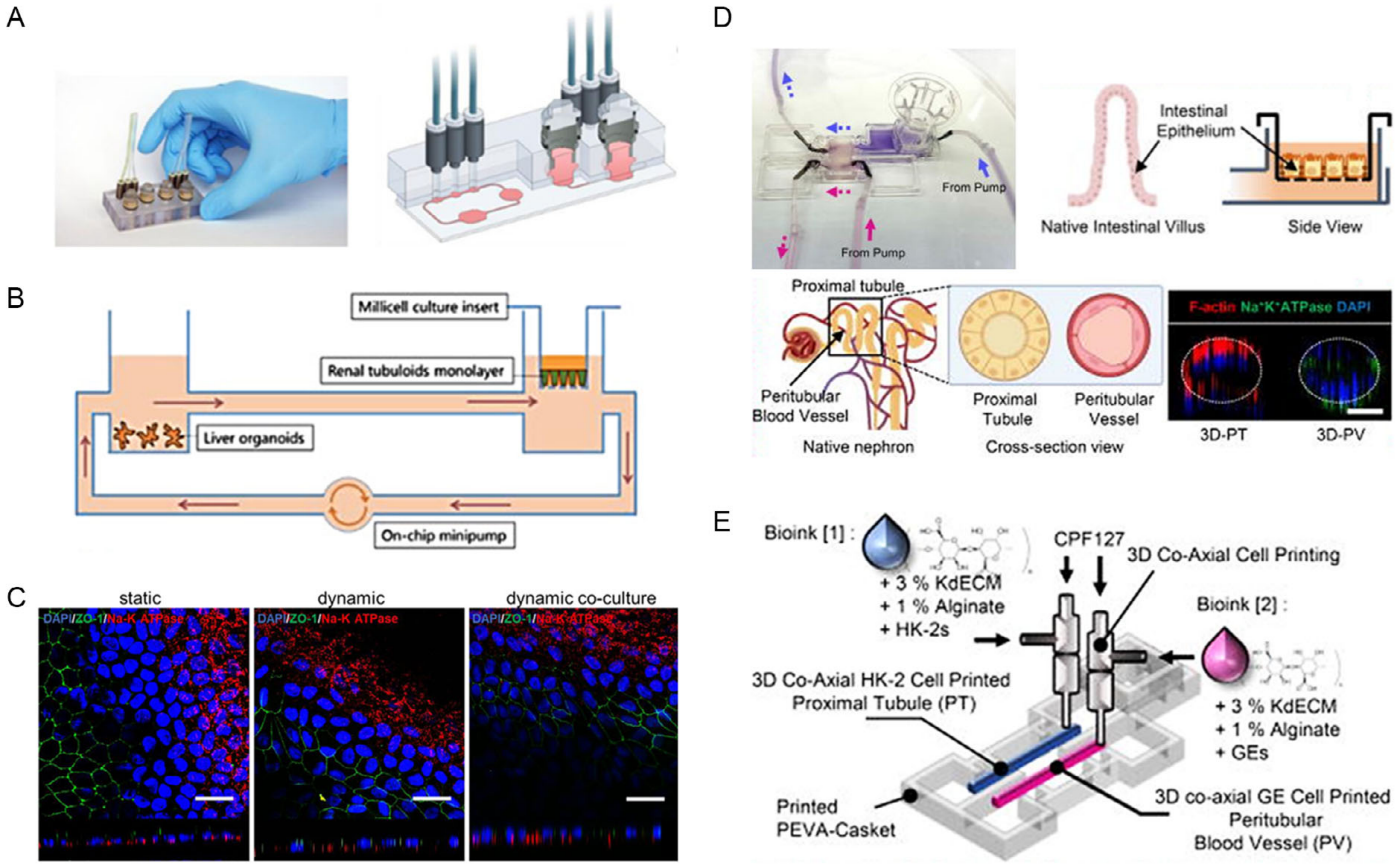
Kidney‐based MOMPS. A) Schematic of MOMPS simulating oxidative kidney injury with liver–kidney crosstalk. B) Operational diagram of MOMPS for modeling oxidative kidney injury. C) Impact of dynamic culture on recovery of renal tubules. Reproduced with permission.^[^ ^183^ ^]^ Copyright 2022, John Wiley and Sons. D) Renal tubule, vascular, and intestine components within MOMPS for modeling secondary hyperoxaluria. E) Schematic of MOMPS simulating secondary hyperoxaluria with intestine–kidney crosstalk. Reproduced with permission.^[^ ^177^ ^]^ Copyright 2022, AIP Publishing.

Nguyen et al. developed a kidney injury MOMPS using human kidney and liver organoids (**Figure** **3**A). They isolated cells from the human kidney tissue to create a renal tubuloid monolayer chamber and a chamber for liver organoids using PDMS, which was then connected to microfluidic channels within a single platform through self‐assembly (Figure 3B). They developed a kidney injury model caused by oxidative stress by flowing hydrogen peroxide through the MOMPS.^[^ ^183^ ^]^ The addition of microfluidic channels made from human mesenchymal stem cell (MSC) and MSC‐derived small extracellular vesicles to the model promoted the restoration of barrier integrity and functional transport, thereby accelerating the recovery of renal tubules after oxidative damage. Furthermore, dynamic culture in microfluidic channels showed that connecting the liver to the kidney through microfluidic channels accelerated the recovery of the renal tubules (Figure 3C). This demonstrates that to accurately mimic multi‐organ kidney diseases in vitro, the design must enable crosstalk between various organs so that physiological substances can function properly. This study is significant because it is the first attempt to fabricate an MOMPS that accurately mimics kidney disease in vitro. However, this model lacks biomaterials capable of replicating each organ‐specific microenvironment, and functional evaluation of the kidney compartment was only briefly conducted, implementing only some of the pathological features of oxidative kidney injury. Nonetheless, this study is a pioneering MOMPS that can contribute to the understanding of the mechanisms and comorbidities of liver and kidney diseases, such as hepatorenal syndrome. Adding organ‐specific biomaterials or nephron function mimetics to the platform can evolve into an advanced model that replicates the complex pathological interactions of liver–kidney diseases.

Yoon et al. developed an MOMPS that mimics the major pathophysiological features of secondary hyperoxaluria, a multifactorial disease in which oxalate absorption is impaired in the intestinal epithelium, leading to kidney stones.^[^ ^177^ ^]^ To mimic kidney disease, it is necessary to replicate the dynamic pathophysiological interactions between the intestinal epithelium and the proximal tubule (Figure 3D). They created a perfusable MOMPS that connects two‐organ mimetics. Using 3D coaxial bioprinting and organ‐derived dECM bioinks that can replicate the microenvironment of each organ, the intestinal epithelium and vascularized proximal tubules were spatially separated (Figure 3E). This model reproduced normal kidney functions such as glucose reabsorption and glomerular filtration barrier formation, and induced disease conditions according to tubular fluid flow dynamics, exhibiting pathophysiological features such as calcium oxalate crystal formation and barrier disruption of the intestinal epithelium and proximal tubules. These features are due to the establishment of dynamically interconnected multi‐organ modules. The model also demonstrated its potential as a drug‐testing platform by dissolving previously formed calcium oxalate crystals when treated with grape seed extract and trisodium citrate, which are known therapies for secondary hyperoxaluria. Despite the significant replication of key features of kidney disease and utilization of various 3D‐bioprinting strategies, the MOMPS design should consider producing a structure that more closely replicates the actual multilayered and complex kidney structure. Moreover, the model does not simulate the multilayered tubular structure, making it challenging to precisely replicate the progression of secondary hyperoxaluria in the vasculature and proximal tubules. Nevertheless, using dECM bioinks and 3D‐bioprinting technologies to replicate the microstructures of the vasculature and proximal tubules, they precisely replicated the pathological mechanisms of multi‐organ diseases and demonstrated their potential as a drug testing platform, contributing to the development of new drugs related to kidney diseases.

Finally, Maschmeyer et al. developed a four‐organ MOMPS designed to model the absorption, distribution, metabolism, and excretion processes in the human body, with a focus on drug testing and systemic toxicity.^[^ ^184^ ^]^ This MOMPS integrated compartments for the intestine, liver, skin, and kidney, simulating the processing mechanisms of drugs and their potential toxic effects across these organs. In this MOMPS, the intestine compartment enables the absorption of orally administered substances, which are then metabolized by hepatocyte spheroids in the liver compartment. The skin compartment represented an alternative absorption route and provided a model for evaluating topical drug effects. The kidney compartment, which mimicked the proximal tubule, played a critical role in excreting drug metabolites. The study highlighted significant organ crosstalk, particularly in the processing and elimination of substances, demonstrating the importance of modeling these interactions for accurate drug testing. The MOMPS was fabricated on a microfluidic chip platform with integrated peristaltic micropumps to maintain fluid flow across the organ compartments, simulating in vivo conditions. The system, which was constructed using PDMS–glass with polymeric membranes to control media flow, maintained organ functionality for up to 28 days, allowing for long‐term studies. The key advantages of this MOMPS include its physiological relevance and ability to enable the study of multi‐organ interactions over extended periods. However, the complexity of the system and the potential issues with PDMS, such as drug absorption, presented challenges that need to be addressed for broader application. Overall, the study demonstrated the potential of MOMPS in providing a comprehensive understanding of drug interactions and toxicity in a more accurate and integrated manner than traditional in vitro models.

Biofabrication technologies, such as organoid fabrication and 3D bioprinting, for developing organ structures for kidney disease, as well as functional biomaterials, such as dECM bioinks, that can replicate the kidney‐specific microenvironment, have been developed.^[^ ^185^ ^]^ However, several kidney disease models require the induction of inflammatory conditions. Therefore, it is necessary to further improve these technologies and biomaterials for inflammatory environments. Lee et al. succeeded in creating a gut–kidney axis on chip, simulating intestinal inflammation caused by *Escherichia coli* and subsequent sequential kidney infection.^[^ ^186^ ^]^ By treating the model with various antibiotics and confirming its responsiveness, they validated the results for clinical applications and functionality. The pathological kidney environment operates dynamically, constantly interacting with more than one organ. Therefore, to accurately replicate pathological functions, it is crucial to combine MOMPS with a dual‐monitoring system capable of simultaneously monitoring the functions and drug responses of the kidneys and other organs.^[^ ^19^ ^]^ Lin et al. developed MOMPS to model drug‐induced nephrotoxicity and hepatotoxicity by integrating liver spheroids and renal proximal tubule barriers.^[^ ^187^ ^]^ It demonstrated significant organ crosstalk, where the liver metabolism of cyclosporine A influenced its toxicity in the kidney. The system enabled real‐time monitoring of drug metabolism and toxicity across organ compartments, maintaining stable liver and kidney functions over extended periods. As a more advanced approach, hydrogel‐based sensors and microfluidic systems must be integrated with MOMPS to enhance multi‐organ interactions and simultaneously identify pathological changes across all relevant organs. In addition, the adoption of advanced automated control systems is essential for obtaining consistent results. Last, because kidney diseases are significantly influenced by genetic factors and intestinal impacts, considering personalized kidney disease, MOMPS models that incorporate intestinal compartments and personalized microbiomes are also crucial.^[^ ^188^ ^]^ The development of control technologies that can effectively utilize patient‐derived stem cells and microbiomes is vital. Fabricating such MOMPS can contribute to the establishment of complex systemic metabolic disease prevention strategies, thereby reducing medical diagnostic costs and improving treatments.^[^ ^19^ ^]^

### 3.3 Diabetes Targeted MOMPSs

Diabetes is a chronic complex disease involving the interaction of multiple organs, such as the pancreas, adipose tissue, liver, and muscles, with no clearly defined pathological mechanism and a highly ambiguous scale for distinguishing between disease prevention and severity of complications.^[^ ^189^ ^]^ Diabetes is characterized by elevated blood glucose levels and vascular dysfunction owing to defects in insulin production and action. The two main types of diabetes are type 1 diabetes, an autoimmune disorder in which the body attacks β cells in the pancreas responsible for insulin production, and type 2 diabetes (T2D), which predominantly affects obese individuals due to insulin resistance and eventual β‐cell dysfunction.^[^ ^190^ ^]^ Most patients have T2D.^[^ ^8^ ^]^ Diabetes is particularly hazardous because of its association with diabetic complications affecting various organs, including the heart, retina, nerves, and skin, with poor prognosis necessitating early diagnosis of diabetic complications.^[^ ^191^ ^]^ Moreover, tailored treatment strategies are required based on disease progression and the occurrence of multi‐organ complications, recognizing that exceptions exist where individuals do not exhibit typical diabetic disease characteristics.^[^ ^192^ ^]^ Current diabetes treatments and insulin injections do not cure the disease, but merely delay its progression, underscoring the urgent need for radical cure research.^[^ ^191^ ^]^ Thus, there is an urgent need to develop precise in vitro diabetes models capable of reproducing diabetic pathologies and microenvironments, enabling research on diabetic mechanisms and testing diabetes treatments.^[^ ^8^ ^]^

Historically, to understand disease trends, research on diabetes and its complications has largely relied on animal experiments involving rodents and sample collection from patients with related diseases. However, animal experiments face challenges in accurately reflecting human pathological ecology owing to interspecies genetic and biochemical metabolic differences, ethical issues, and difficulties in analyzing real‐time physiological environmental changes.^[^ ^193^ ^]^ Therefore, to represent diabetes pathology biochemically, various in vitro models utilizing microfluidic‐based microelectromechanical systems (MEMSs) or hydrogel‐based bioinks for the upward stacking of cells using 3D‐bioprinting technologies have been developed. Organs directly associated with diabetes include the pancreas, liver, adipose tissue, and blood vessels, and single‐organ models mimicking these organs have been primarily developed for diabetes research.^[^ ^8^ ^]^ For example, Lee et al. developed an MOMPS, fabricated with PDMS microfluidics, to model glucose metabolism, integrating pancreas, muscle, and liver compartments to simulate T2D.^[^ ^194^ ^]^ Significant organ crosstalk was observed, where pancreatic insulin influenced muscle glucose uptake and liver glucose homeostasis. This MOMPS offers physiological relevance but faces challenges in terms of scaling and using animal cell lines. Existing models frequently encounter limitations such as issues with cell selection or being able to simulate only certain aspects of diabetes pathology, such as inflammation. Additionally, they usually lack the ability to replicate the microenvironment of each organ adequately, making them insufficient to fully represent the complex pathophysiology of diabetes as it occurs in humans due to multi‐organ interactions.^[^ ^195^ ^]^

Therefore, the successful development of in vitro diabetes models requires the use of biomaterials capable of replicating diabetes‐specific pathological microenvironments and modeling multi‐organ interactions related to diabetes within a single platform.^[^ ^193^ ^]^ Additionally, it is crucial to fabricate vascular structures within the platform to implement complex interorgan interactions via blood vessels and simultaneously incorporate hyperglycemia and inflammatory environments that are essential for simulating diabetes pathology.^[^ ^8^ ^]^ Efforts to address these challenges include the development of biomaterials capable of replicating organ‐specific microenvironments and biofabrication technologies that enable their integration into platforms.^[^ ^18^ ^]^ Based on these technologies, ongoing efforts are advancing toward the creation of MOMPS capable of integrating multiple organs related to diabetes within a single platform. The excellence and production technologies of the latest diabetes mimetic MOMPS are illustrated through various examples of multi‐organ in vitro platforms presented later.

Essaouiba et al. developed a foundational pancreas–liver organ‐on‐chip model to simulate the interactions related to T2D.^[^ ^196^ ^]^ This system integrates two key compartments. One part is the pancreas, which is represented by rat islets of Langerhans responsible for insulin production, and another part is the liver, which is represented by rat hepatocytes essential for glucose metabolism. The model effectively simulated the dysregulation of glucose homeostasis, which is a core feature of T2D, focusing on the impact of insulin resistance on organ function. Significant organ crosstalk was observed within this MOMPS, with pancreatic insulin secretion aiding in restoring impaired liver functions, such as albumin production and the expression of liver markers such as cytochrome P450, family 3, subfamily a, polypeptide 2 (CYP3A2), and cytokeratin 18 (CK18). Additionally, the liver influenced pancreatic islets by modulating the insulin‐related gene expression, underscoring the crucial role of pancreas–liver communication in maintaining glucose homeostasis. This MOMPS is constructed on a platform composed of PDMS layers with collagen‐coated surfaces to facilitate hepatocyte adhesion. The platform enables long‐term coculture and precise microenvironment control, which are essential for studying chronic diseases such as T2D. Despite these advantages, the model's complexity and the limitations associated with using rat cells present challenges in directly translating the findings to human physiology. Nonetheless, this study demonstrated the potential of pancreas–liver organ‐on‐chip models as pioneering tools that offer valuable insights into the interactions between these critical organs and advance our understanding of T2D.

**Figure 4:**
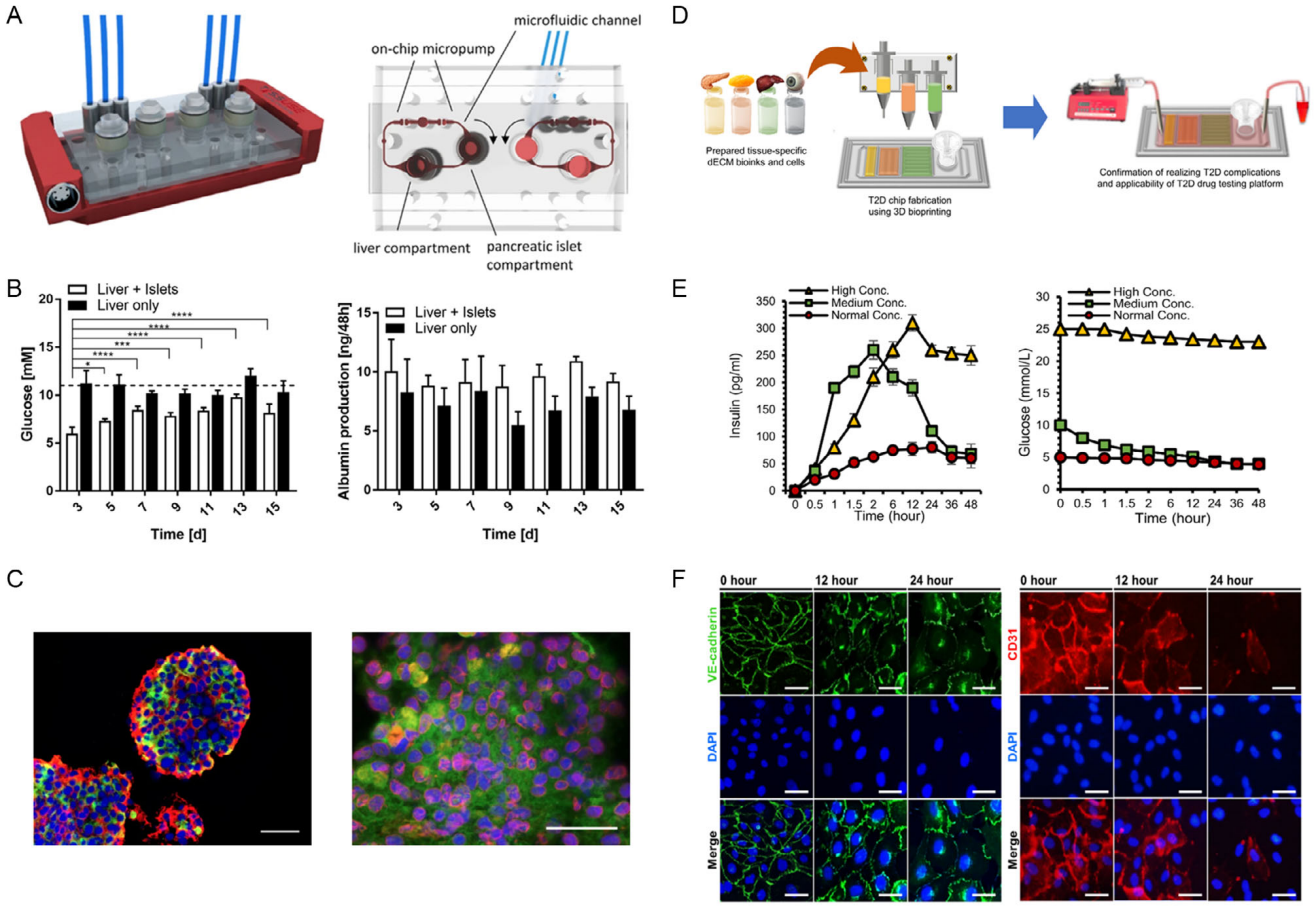
Diabetes‐targeted MOMPS. A) Schematic of MOMPS simulating diabetes with liver‐pancreas crosstalk. B) Effect of liver–pancreas crosstalk on liver function and blood glucose regulation. C) Impact of coculture on the function of pancreatic compartment (left) and liver compartment (right). Reproduced with permission.^[^ ^197^ ^]^ Copyright 2018, Springer Nature. D) Schematic of MOMPS simulating diabetes with pancreas–visceral adipose tissue–liver–retina crosstalk. E) Insulin and glucose level control in MOMPS simulating diabetes. F) Disruption of vascular endothelial barrier within MOMPS under diabetic condition. Reproduced with permission.^[^ ^8^ ^]^ Copyright 2023, John Wiley and Sons.

Bauer et al. created a foundational T2D mimetic MOMPS capable of simulating the effects of glucose on insulin secretion by connecting chambers for human liver spheroids composed of a human liver epithelial cell line and stellate cells, as well as chambers for human pancreatic islet organoids, using PDMS (**Figure** **4**A). They were integrated into a single platform via microfluidic channels by self‐assembly.^[^ ^197^ ^]^ A T2D environment was simulated by increasing the glucose concentration in the medium flowing through the microfluidic channels to 11 mM. The authors demonstrated that connecting the liver and pancreas via microfluidic channels within the MOMPS resulted in more pronounced increase in insulin secretion and decrease in glucose concentration compared with operating each part separately (Figure 4B,C). This underscores the need for organs associated with diabetes to interact for hormones and enzymes to function properly, proving the concept of modeling diabetes, a disease that spans multiple organs in vitro. This study was a significant initial attempt to create an in vitro model capable of simulating diabetes, specifically related to complications involving the pancreas and liver, contributing to the understanding of the mechanisms and concurrent conditions associated with diabetes‐related pancreatic and hepatic complications. However, this model lacks designs to implement organ‐specific microenvironments and the use of biomaterials, focusing solely on the liver and pancreatic compartments, thereby limiting its ability to simulate certain aspects of diabetes. Despite these limitations, this study suggests the potential for developing an advanced model capable of simulating complex physiological interactions between organs by adding organ‐specific biomaterials, primary cells, and vascular mimetics to the platform.

Kim et al. utilized a 3D bioprinter equipped with a multi‐head nozzle to incorporate tissue‐specific dECM bioinks from various organs, enabling the one‐step fabrication of a T2D mimetic MOMPS within a single platform (Figure 4D). This platform can simultaneously simulate hyperglycemic and inflammatory environments.^[^ ^8^ ^]^ They designed separate compartments within the MOMPS for the pancreatic, adipose tissue, and liver regions, and introduced a retinal compartment to induce applications related to T2D complications. Moreover, they positioned a monolayer of human umbilical vein endothelial cells on the upper part of the organ sections and covered them with PDMS to incorporate rectangular vessels within the MOMPS, connecting all multi‐organ compartments. The accumulation of visceral adipose tissue exacerbates inflammatory conditions and increases the prevalence of diabetes.^[^ ^198^ ^]^ This study is significant as it is the first attempt to mimic a series of pathophysiological processes related to obesity‐associated diabetes using a visceral adipose tissue‐derived dECM bioink and macrophages within an MOMPS. MOMPS was meticulously designed to simulate the precise aspects of diabetes, considering the arrangement and compartmentalization of organs, effects of multi‐organ crosstalk, and vascularization. Additionally, it effectively mimicked several hallmark features of diabetes, such as insulin resistance, hyperglycemia, increased inflammatory cytokines, and breakdown of the blood–retinal barrier, enabling advanced modeling of diabetic complications and testing of various diabetes therapies (Figure 4E,F). However, this model has limitations in fully implementing the mechanisms of diabetes owing to the absence of muscle and organ compartments capable of glucose consumption. Furthermore, although the possibility of modeling retinal complications has been confirmed, additional validation is required to assess its applicability to other complications. In addition, it cannot precisely simulate the extent of vascular damage associated with the progression of diabetes because of its inability to replicate multilayered vascular structures. Despite these limitations, leveraging biomaterials capable of mimicking hyperglycemic and inflammatory environments and utilizing 3D‐bioprinting technology allow for the precise and rapid implementation of diabetic pathophysiology, making it a valuable platform for testing diabetes therapies aimed at radical cure development.

Developed diabetes models have utilized biofabrication technologies such as self‐assembly, organoid production, and 3D bioprinting using dECM derived from various organs, including visceral adipose tissue, enabling the creation of diabetes‐specific microenvironments. However, several aspects require further improvement and application. Ronaldson‐Bouchard et al. recently aimed for more precise organ‐to‐organ crosstalk by differentiating stem cells for each organ compartment, achieving long‐term maturation without immune rejection and functionality akin to that of native organs.^[^ ^199^ ^]^ These compartments are interconnected within vascularized structures with vascular barriers. Although this approach achieves the assembly of diverse organ compartments within a single platform to accurately reflect organ‐to‐organ crosstalk, challenges remain, including contamination during self‐assembly and achieving leak‐free compartment integration. Furthermore, attempts to apply this method to various diabetic complications beyond conventional diabetes MOMPS are limited. Hence, the development of new biofabrication technologies is essential, allowing for the creation of diverse organ compartments as needed and their direct integration into platform vascular structures.^[^ ^22^ ^]^ Additionally, to accurately simulate dynamic environments necessitating constant communication between multiple organs, chamber‐mimicking factors such as fluid dynamics and mechanical stresses integrated with diabetes‐mimetic MOMPS can enhance model reliability.^[^ ^200^ ^]^ Research on advanced hydrogels, such as diabetic patient‐derived dECM bioinks, that effectively replicates the pathophysiological environments of diabetes, is crucial.^[^ ^8^ ^]^ Furthermore, the adoption of automated biofabrication technologies and advanced control systems is imperative for achieving efficient and consistent outcomes.^[^ ^122^ ^]^ Finally, given the variability in diabetes owing to genetic and lifestyle factors, personalized diabetes MOMPS models are essential.^[^ ^191^ ^]^ The effective utilization of patient‐derived stem cells in fabrication technologies is crucial for developing personalized ex vivo models, contributing to tailored therapeutic and preventive strategies, and significantly reducing preclinical and clinical costs.

### 3.4 Other Organ‐Targeted MOMPSs

In addition to the previously mentioned diseases, notable complex disorders also include those targeting the liver or gut, which play a direct role in systemic metabolism and are closely interconnected with other organs.^[^ ^201^, ^202^ ^]^ The liver, as a central hub for metabolism, detoxification, and immune responses, and the intestine, which is responsible for nutrient absorption, barrier function, and housing the gut microbiota, interact with other organs and frequently contribute to the development of complex systemic diseases.^[^ ^201^, ^202^ ^]^ These contributions are crucial in diseases such as psoriasis and systemic inflammatory conditions, where the liver or gut play significant roles. Especially, the liver plays a critical role in drug metabolism, significantly influencing other organs, such as the skin, through organ crosstalk.^[^ ^203^ ^]^ Utilizing dynamic microfluidic platforms and 3D liver cultures to simulate physiologically relevant environments for drug testing, these systems are designed to maintain liver functionality over extended periods.^[^ ^204^ ^]^ The inclusion of a liver compartment is essential for the development of these systems, as it ensures accurate modeling of drug‐induced effects on other tissues, with several examples demonstrating its necessity.^[^ ^203^, ^205^ ^]^ Recent advancements in MOMPS technologies, including microfluidic platforms and 3D bioprinting, have been designed to accurately replicate the complex microenvironments and dynamic interactions between these organs, enabling the study of complex diseases and testing therapies under more physiologically relevant settings.^[^ ^18^ ^]^ This section focuses on MOMPS targeting the liver and gut, in addition to those related to the brain, kidney, and diabetes, aiming to enhance our understanding of the mechanisms underlying systemic diseases and to provide new therapeutic insights through these models.

Lee et al. investigated the gut–skin axis using an MOMPS that connected the gut and skin compartments.^[^ ^206^ ^]^ This system was particularly focused on modeling inflammatory skin diseases such as psoriasis, highlighting the critical crosstalk between the gut and the skin. In this model, two key organ compartments were involved: the gut, represented by Caco‐2 epithelial cells and RAW 264.7 macrophages, and the skin, represented by HaCaT keratinocytes and primary dermal fibroblasts. The gut and skin compartments were initially cultured separately and then connected via a microfluidic platform that facilitates the flow of fluids and signaling molecules between the two. The study revealed significant organ crosstalk, primarily through inflammatory responses. Gut inflammation, induced by cytokines such as tumor necrosis factor‐α (TNF‐α), leads to systemic inflammation that negatively impacts skin health, increasing inflammatory markers and reducing skin cell viability. This mimics the conditions observed in inflammatory skin diseases where gut health directly influences skin pathology. Additionally, the disruption of the gut barrier, akin to conditions such as the leaky gut syndrome, allows inflammatory molecules to translocate to the skin, further compromising the skin barrier function. The study also highlighted metabolic crosstalk, where gut‐derived metabolites such as fatty acids exacerbate skin inflammation. This gut–skin chip model provided a valuable tool for studying the complex interactions between the gut and the skin, offering insights into the systemic nature of diseases such as psoriasis and highlighting the importance of maintaining gut health for overall skin wellness. Despite the system's complexity and the challenges associated with maintaining long‐term stability, it presented a promising approach to understanding and treating systemic inflammatory diseases.

Wagner et al. focused on developing an MOMPS designed to model drug‐induced toxicity and organ interactions, specifically between the liver and skin.^[^ ^205^ ^]^ The system was used to study the effects of troglitazone, a drug known for its hepatotoxicity, on both liver and skin tissues. This MOMPS integrated two key compartments. The liver compartment comprises human liver microtissues, including hepatic cells, and a skin compartment consists of various skin cell types cultured at an air–liquid interface (ALI). The study observed significant organ crosstalk, where metabolites produced by the liver affected the skin's health and functionality. The system employed a microfluidic platform with a peristaltic micropump to simulate blood circulation and used PDMS and glass substrates for construction. Key advantages of this MOMPS included its ability to maintain stable cocultures over extended periods, providing a more physiologically relevant model for drug testing. However, challenges such as the complexity of maintaining precise culture conditions and potential material limitations, such as PDMS's tendency to absorb small molecules, were noted. Overall, the study highlighted the potential of dynamic multi‐organ chips in advancing preclinical drug testing and personalized medicine by accurately simulating organ interactions and systemic effects.

Reinhold et al. focused on modeling lung–liver interactions during respiratory infections using an MOMPS that connected the lung and liver compartments.^[^ ^207^ ^]^ This system specifically simulated the systemic effects of lung infections on liver function, emphasizing crosstalk between these organs during disease states such as pneumonia and chronic obstructive pulmonary disease. In this MOMPS, two key organ compartments were integrated: the lung module, which utilizes primary human bronchial epithelial cells or alveolar type cells cultured at the ALI to emulate the lung environment, and the liver module, which employed the Huh‐7 cell line to model hepatic function. The Quasi Vivo system, a microfluidic platform, facilitates fluid flow between these compartments, enabling the exchange of inflammatory mediators. Significant organ crosstalk was observed in this model. Upon bacterial infection of the lung compartment with pathogens such as *Haemophilus influenzae* and *Pseudomonas aeruginosa*, inflammatory cytokines such as TNF‐α, monocyte chemotactic protein‐1, and macrophage inflammatory protein‐3 were released. These cytokines circulated to the liver compartment, inducing transcriptomic changes in liver cells. This finding demonstrates how lung infections can trigger systemic inflammatory responses that impact liver function, providing critical insights into the lung‐liver axis during infection. Key features of this MOMPS include its realistic simulation of infection and systemic interaction, offering a more accurate model for studying the effects of lung infections on liver function. However, challenges remain, such as the complexity of maintaining different culture conditions and the absence of other relevant cell types, such as fibroblasts and endothelial cells. Despite these hurdles, the study underscored the potential of MOMPS in advancing our understanding of organ interactions in disease and in developing targeted treatments.

De Mello et al. developed an MOMPS to simulate the systemic effects of drugs, particularly focusing on the interactions between the heart and liver following topical drug application.^[^ ^208^ ^]^ The MOMPS integrated three organ compartments: a heart module using human iPSC‐derived cardiomyocytes to assess cardiac function, a liver module using human primary hepatocytes to model hepatic activity, and a skin module employing a synthetic membrane to simulate human skin. The study revealed significant organ crosstalk, where the application of drugs such as diclofenac and ketoconazole on the skin led to systemic effects that impacted both cardiac and liver functions. For example, diclofenac decreased cardiac conduction velocity and contractile force while altering liver enzymatic activity. The system employs a microfluidic platform made from poly(methyl methacrylate) and PDMS, enabling controlled fluid flow and recirculation between compartments, and integrates MEMS and membrane electrode assembly to monitor cardiac activity in real time. The key advantages of this MOMPS included its ability to simulate realistic drug‐delivery scenarios, multi‐organ interactions, and the potential for chronic and acute toxicity testing. However, challenges include complexity of the system, requirement of precise control of experimental conditions, and limitations related to the synthetic skin surrogate, which may not fully replicate the complexity of human skin. This innovative MOMPS represents a significant advancement in predicting human drug responses, particularly for understanding the systemic effects of topically applied drugs, offering a more comprehensive model for drug safety evaluations.

McAleer et al. focused on a more specific MOMPS that connects the heart and liver compartments to study the pharmacokinetic–pharmacodynamic relationships of drugs, particularly in the context of cardiotoxicity.^[^ ^209^ ^]^ Their system effectively simulated the on‐target efficacy and off‐target toxicity of drugs, including their metabolites, demonstrating the potential of MOMPS to predict human responses more accurately. The organ crosstalk observed in this system, particularly between the heart and the liver, highlighted the importance of considering metabolic processes in drug safety assessments. The use of a reconfigurable, pumpless system enables the study of real‐time functional outcomes, such as cardiac electrical and mechanical responses, further underscoring the utility of MOMPS in preclinical drug evaluation. However, these results also revealed the technical challenges that must be addressed for broader application, such as the need for precise control of culture conditions and the limitations of current biomaterials. Despite these challenges, the advancements represented by these studies provide a promising outlook for the future of drug discovery and disease modeling.

Liver‐ and gut‐targeted MOMPSs are designed to simulate systemic complex diseases by enabling the coculture of multiple cell types within a controlled environment that integrates fluid flow and mechanical forces to replicate in vivo conditions.^[^ ^19^ ^]^ These models offer significant advantages, such as enhanced physiological relevance and the ability to enable the study of interorgan communication. However, they also present challenges, including the complexity of system integration and the need for precise control over experimental conditions.^[^ ^96^ ^]^ Despite the technical difficulties associated with integrating multiple organ systems and maintaining long‐term stability, advancements in MOMPS technology hold great promise for improving disease modeling and accelerating drug discovery.^[^ ^208^ ^]^

## 4 Conclusions and Future Perspectives

The MOMPS, capable of simulating interactions between various organs, offer a promising new approach for studying systemic metabolic disorders. The advent of MOMPS signifies a significant shift from conventional animal models and single‐organ systems to more advanced ex vivo systems that better mimic human physiology.^[^ ^19^ ^]^ MOMPS provide a more accurate and comprehensive model for studying the complex interactions that contribute to these diseases by replicating human microphysiology and simulating interorgan crosstalk.^[^ ^20^ ^]^ These systems offer substantial advantages over conventional in vitro models, including the ability to study controlled interactions between multiple organs and the scalability to represent the complexity of human physiology. Careful selection of appropriate biomaterials and model fabrication methods is crucial, to simulate 3D structure of disease model and specific pathological microenvironments.^[^ ^16^ ^]^ In this regard, advanced biofabrication techniques such as functional biomaterials, including dECM, 3D bioprinting, and organoid production, have been developed, enabling the processing of diverse cells within MOMPS into pathophysiological structures suitable for study.^[^ ^18^ ^]^ In conclusion, MOMPS effectively mimic human physiology and various systemic metabolic diseases, thereby enhancing the understanding of disease mechanisms and serving as a highly accurate drug‐testing platform to improve efficiency and predictive capability in preclinical research. However, the widespread application of MOMPS in disease modeling is constrained by several challenges, including the need for sophisticated engineering and standardized protocol development, which are discussed alongside proposed approaches to overcome these issues. In addition, ethical and legal considerations related to MOMPS fabrication are briefly addressed.

Metabolic disorders develop over extended periods, making it crucial for MOMPS to maintain the durability and stability of their physiological functions.^[^ ^210^ ^]^ To achieve this goal, innovations in biomaterials, biofabrication, and monitoring technologies are required. First, biological materials suitable for MOMPS fabrication must possess specific physical properties to ensure the mechanical stability of the constructed structures.^[^ ^89^ ^]^ For instance, hydrogels have limited application as bioinks because of restricted cross‐linking, which limits their potential for MOMPS fabrication. Therefore, the development of advanced biomaterials capable of controlling their properties through chemical and physical treatments of existing biomaterials is necessary to enhance the design flexibility in MOMPS fabrication. The dECM, which is known to simulate organ‐specific microenvironments, lacks mechanical strength despite its biochemical excellence, presenting challenges for producing high‐resolution structures. One approach to address these limitations involves integrating hydrogels with polymers, such as PCL, to enhance their structural integrity.^[^ ^211^ ^]^ However, plastic scaffolds may restrict the use of printed replicates in native tissue replacement. Enhancing the mechanical properties of weak biomaterials through integration with other hydrogels is a rational strategy for improving biomaterial utility through physical property enhancement.^[^ ^71^ ^]^ Ali et al. used methacrylated‐dECM bioinks to enhance their physical properties and applied them to kidney regeneration.^[^ ^212^ ^]^ Another method involves enhancing the hydrogel properties through integration with photopolymerizable substances, as demonstrated by Kim et al. who successfully created centimeter‐scale structures using photopolymerizable dECM bioinks via extrusion‐based 3D bioprinting.^[^ ^90^ ^]^ Another approach involves creating a support bath using hydrogel, printing dECM structures within the bath, and subsequently removing the support bath. This method, known as embedded printing, is particularly suitable for fabricating large‐scale structures.^[^ ^213^ ^]^ Kong et al. successfully utilized cartilage‐derived dECM bioink and embedded printing to fabricate a volumetric vitreous body construct.^[^ ^213^ ^]^ These methods can maintain the tissue‐specific microenvironment simulation capabilities of dECM bioinks while improving their mechanical properties to fabricate stable 3D structures in MOMPS. Additionally, advancing biofabrication is crucial for the efficient development of various disease models using these biomaterials, focusing on precision and reducing fabrication time. This advancement can be achieved by combining existing technologies. For example, Brassard et al. successfully combined extrusion‐based and inkjet bioprinting technologies to create large tissue blocks within an MOMPS.^[^ ^214^ ^]^ Furthermore, the precision of hydrogel‐based MOMPS fabrication has historically been limited by challenges in the real‐time control of fabrication environments, such as humidity and temperature. Thus, it is necessary to establish a hydrogel‐based biofabrication technology database and apply real‐time automated control systems to fabrication systems.^[^ ^215^ ^]^ The integration of MOMPS with computational models and various sensors can enhance the predictability and utility of disease modeling. Since metabolic disorders often involve crosstalk among multiple organs, the simultaneous detection of abnormalities in each organ is crucial. Therefore, deploying sensors capable of detecting organ‐specific abnormalities within MOMPS and integrating them with computational models can help analyze the complex physiological data generated by MOMPS, predict disease progression, and effectively assess treatment outcomes.^[^ ^216^ ^]^

Another challenging aspect of developing MOMPS is the need for sophisticated bioengineering and precise control of microenvironments to ensure the proper functioning of multiple organ systems and their appropriate interactions.^[^ ^4^ ^]^ Therefore, standardization and reproducibility of MOMPS are essential for widespread adoption, requiring these fabrication systems to be replicable and generate consistent results across various laboratories.^[^ ^89^ ^]^ For example, variations in the composition of natural biomaterials and dECM can lead to functional differences in each batch. Addressing these issues necessitates standardization of the preparation of biomaterials, such as batch production and mixing methods. Furthermore, quantitative standardization based on the analysis of biomaterial components allows for more robust use. In addition, animal‐derived biomaterials must undergo a uniform evaluation for immunogenicity removal in terms of biocompatibility.^[^ ^11^ ^]^ Challenges also arise from the use of toxic chemicals in biomaterial preparation, which can affect cell survival and lead to incomplete cell removal, resulting in heterogeneous immune responses. To mitigate these issues, it is essential to fundamentally analyze how preparation processes affect the composition of ECM components in biomaterials and adopt methods that preserve ECM components to the fullest extent possible. Wang et al. utilized supercritical carbon dioxide in the preparation process to minimize the ECM damage.^[^ ^217^ ^]^ Moreover, advanced rapid detection methods should be developed to verify the absence of residual toxicities and cellular components in dECM, along with the implementation of standardized quality control.^[^ ^89^ ^]^ Thus, through the development of standardized protocols and validation criteria, the aforementioned issues can be effectively addressed, enabling the fabrication of precise disease‐mimicking MOMPS using a wide range of biomaterials and biofabrication methods.

Finally, ethical and legal issues must be addressed to effectively utilize the various biomaterials and biofabrication technologies for MOMPS fabrication. The development of diseases that mimic MOMPS in various organs has the potential to overcome the ethical challenges associated with indiscriminate animal experimentation, clinical trials, and organ transplantation.^[^ ^218^ ^]^ However, there is currently no comprehensive global discussion on the ethical and legal issues related to MOMPS. The MOMPS fabrication involves the use of various human‐ and animal‐derived materials and raises interdisciplinary issues spanning biology, bioethics, and philosophy.^[^ ^218^ ^]^ Some emerging ethical and legal issues associated with MOMPS include the following.

The origin of the biomaterials and cells used in MOMPS fabrication is a critical ethical consideration.^[^ ^218^ ^]^ Ethical principles and regulations are necessary to obtain and utilize human and animal tissues and cells. This includes aspects such as tissue and cell donation, consent, and the protection of genetic information. Furthermore, a thorough research and validation of the safety and risks of the biomaterials used are essential. The use of unverified materials or technologies that pose risks to patients should be avoided. Second, there are issues related to consent and personal data protection. In MOMPS fabrication technologies such as 3D bioprinting, digital 3D models represent personalized human data.^[^ ^219^ ^]^ Therefore, the protection of personal information should be considered in the context of data protection laws. Such personal information must be securely protected to prevent unauthorized access and misuse. Moreover, obtaining prior consent is crucial when harvesting cells and tissues from patients or donors. Obtaining informed consent ensures that individuals fully understand how their cells will be used, the scope of the research, and the potential impacts.

In research and medical fields involving the use of animal‐ or human‐derived biomaterials, ethical approval from ethics committees for research or clinical trials is required, and there is a need to harmonize these protocols globally.^[^ ^218^ ^]^ Currently, there is no unified law regarding these matters. Therefore, regulatory agencies such as the USFDA and European Medicines Agency (EMA) need to expedite the establishment of unified laws to ensure the safety and ethical treatment of research participants. For example, the EMA's Gene Therapy Act categorizes certain tissue‐engineered products as advanced therapy medicinal products (ATMPs) and regulates them accordingly.^[^ ^220^ ^]^ Reference to such laws could facilitate the creation of unified laws, given the similarity in purpose between MOMPS and ATMPs. Furthermore, to verify the safety and efficacy of MOMPS and ensure product quality, the production, sale, and use of various biomaterials and MOMPS must comply with local laws and regulations. Additionally, the development of MOMPS typically involves various stakeholders, including researchers, institutions, companies, and hospitals, which may lead to issues related to patents and copyrights. Therefore, establishing clear intellectual property and respecting rights are essential to ensure a fair distribution of benefits among contributors and promote collaboration.^[^ ^221^ ^]^

In conclusion, various biomaterials and biofabrication technologies hold promise for widespread application in the development of disease‐mimicking and patient‐specific MOMPS by implementing organ‐specific microenvironments and organ interactions. This approach offers a new avenue for studying the pathological mechanisms of systemic metabolic diseases, shortening the duration of clinical trials and developing new drugs.

## Conflict of Interest

The authors declare no conflict of interest.
